# The changing risk of *Plasmodium falciparum* malaria infection in Africa: 2000–10: a spatial and temporal analysis of transmission intensity

**DOI:** 10.1016/S0140-6736(13)62566-0

**Published:** 2014-05-17

**Authors:** Abdisalan M Noor, Damaris K Kinyoki, Clara W Mundia, Caroline W Kabaria, Jonesmus W Mutua, Victor A Alegana, Ibrahima Socé Fall, Robert W Snow

**Affiliations:** a Malaria Public Health Department, Kenya Medical Research Institute-Wellcome Trust Research Programme, Nairobi, Kenya; b Centre for Tropical Medicine, Nuffield Department of Clinical Medicine, University of Oxford, Oxford, UK; c WHO, Regional Office for Africa, Brazzaville, Republic of Congo

## Abstract

**Background:**

Over a decade ago, the Roll Back Malaria Partnership was launched, and since then there has been unprecedented investment in malaria control. We examined the change in malaria transmission intensity during the period 2000–10 in Africa.

**Methods:**

We assembled a geocoded and community *Plasmodium falciparum* parasite rate standardised to the age group 2–10 years (*Pf*PR_2–10_) database from across 49 endemic countries and territories in Africa from surveys undertaken since 1980. The data were used within a Bayesian space–time geostatistical framework to predict *Pf*PR_2–10_ in 2000 and 2010 at a 1 × 1 km spatial resolution. Population distribution maps at the same spatial resolution were used to compute populations at risk by endemicity class and estimate population-adjusted *Pf*PR_2–10_ (PA*Pf*PR_2–10_) for each of the 44 countries for which predictions were possible for each year.

**Findings:**

Between 2000 and 2010, the population in hyperendemic (>50% to 75% *Pf*PR_2–10_) or holoendemic (>75% *Pf*PR_2–10_) areas decreased from 218·6 million (34·4%) of 635·7 million to 183·5 million (22·5%) of 815·7 million across 44 malaria-endemic countries. 280·1 million (34·3%) people lived in areas of mesoendemic transmission (>10% to 50% *Pf*PR_2–10_) in 2010 compared with 178·6 million (28·1%) in 2000. Population in areas of unstable or very low transmission (<5% *Pf*PR_2–10_) increased from 131·7 million people (20·7%) in 2000 to 219·0 million (26·8%) in 2010. An estimated 217·6 million people, or 26·7% of the 2010 population, lived in areas where transmission had reduced by at least one *Pf*PR_2–10_ endemicity class. 40 countries showed a reduction in national mean PA*Pf*PR_2–10_. Only ten countries contributed 87·1% of the population living in areas of hyperendemic or holoendemic transmission in 2010.

**Interpretation:**

Substantial reductions in malaria transmission have been achieved in endemic countries in Africa over the period 2000–10. However, 57% of the population in 2010 continued to live in areas where transmission remains moderate to intense and global support to sustain and accelerate the reduction of transmission must remain a priority.

**Funding:**

Wellcome Trust.

## Introduction

The Roll Back Malaria initiative was launched in 1998^1^ at a time when Africa was grappling with an unprecedented disease epidemic.^2^ Increases in overseas development assistance have led to substantial improvements in the number of vulnerable populations protected against malaria infection and who have access to drugs that effectively treat the disease.^3^, ^4^ However, for most malaria-endemic countries in Africa, the disease burden associated with *Plasmodium falciparum*, and how this has changed over the decade 2000–10, remains poorly defined.

Attempts to track the changing burden of malaria in Africa have focused on modelled predictions of clinical and fatal outcomes.^4^, ^5^, ^6^, ^7^, ^8^, ^9^ However, the clinical presentation of *P falciparum* shares similar symptoms with competing causes of febrile illness and death, the presence of infection does not infer disease due to acquired clinical immunity, and conversely malaria infection might be an underlying risk factor for deaths from other causes. Most importantly, disease and mortality events, directly or indirectly associated with malaria infection, are treated and occur outside of the formal health sector and are thus under the radar of poorly functioning health information and civil registration systems.

A less ambiguous and ubiquitous measure of malaria, which has been used for over 100 years in Africa,^2^ is whether an individual selected through random community surveys has evidence of infection after a blood sample examination. Infection prevalence has been used since the 1950s to define categories of endemic risk to guide and monitor progress towards malaria elimination targets.^10^, ^11^ Infection prevalence has a predictable relation to other, less frequently measured, parameters of transmission intensity and has been used to model control timelines to reduction of transmission with different combinations of intervention^12^, ^13^ and in the decision pathway to predict the likelihood of elimination.^14^

Recent modelled descriptions of parasite prevalence in Africa predict the contemporary intensity of *P falciparum*.^15^, ^16^ These predictions do not provide information on the extent and intensity of transmission before scaled malaria control. Consequently, they cannot be used to examine changing risks, the effect of individual or combined interventions, or the rebound transmission that might be expected if financing for malaria control were to end.

Herein, we present a temporal and spatial analysis of the largest parasite prevalence data assembly in Africa to define *P falciparum* transmission intensity at the launch of Roll Back Malaria in 2000 and a decade later after substantial financing of malaria control.

## Methods

### Identification of the limits of unstable and stable *P falciparum* transmission

We used combinations of medical intelligence, reported case incidence, and extreme climatic conditions to define (a) areas of Africa where *P falciparum* has been eliminated or where low ambient temperature does not allow the parasite to survive long enough in the mosquito vector to maintain human transmission; (b) unstable transmission areas where documented case incidence is less than 1 autochthonous clinical case per 10 000 population per year within a defined geographical area or where extreme aridity—defined from remotely sensed correlates of moisture—probably restricts adult and larval vector survival to localised transmission sites where standing water has been artificially created; and (c) stable transmission areas represented by suitable climatic conditions for transmission and where case incidence is likely to be at least 1 clinical case per 10 000 population per year. The appendix provides full details of data sources and methods used to define the spatial extents of malaria-free, unstable transmission, and stable transmission areas in 2000 and how these margins changed by 2010.

### Parasite prevalence survey data assembly

We undertook a cascade search between January, 2005, and July, 2013, to identify all possible survey sources, undertaken since January 1980, in which individuals were examined for peripheral blood-stage *Plasmodium* infection at one timepoint. We searched electronic peer-reviewed journals, unpublished reports from European and African academic archives, national ministry of health archives, unpublished data requests from the pan-African research community and the assembly of national household malaria indicator, and nutritional and school health surveys. The longitude and latitude of each survey location was established by global positioning systems, digital place name gazetteers, and remotely sensed maps. The community *Plasmodium falciparum* parasite rates were standardised to the age group 2–10 years (*Pf*PR_2–10_) using an algorithm described elsewhere.^17^ Data were excluded if the survey location could not be positioned, the area surveyed exceeded 5 km^2^, the area was located on small offshore islands where attribute data such as climatic covariates could not be reconciled, the sample surveyed was fewer than 15 persons, or the first of repeat surveys in the same population could not be uniquely identified. The appendix provides a detailed description of the data search, assembly, geocoding, age correction, and country-level data exclusions and inclusions.

### Modelling predicted *P falciparum* infection prevalence in 2000 and 2010

The survey data were unevenly distributed in space and time. However, the spatial and temporal dependencies of the data within countries enabled the application of model-based geostatistical methods that interpolate from data at known locations and time to provide predictions of quantities and estimates of their uncertainty at locations and times where data do not exist.^18^ We elected to analyse data per country, rather than at continental or subregional levels. This factor is an important distinction to previous efforts,^15^, ^16^ and we accept that borrowing information from a country that has implemented substantial malaria control interventions and is data rich to make predictions in its neighbouring country with less control or data can be misleading.

Within each country, we used information from the available age-corrected survey data (sample size and numbers positive) at known locations (longitude and latitude) and times (year) with a minimal set of conservative, long-term climate and human settlement covariates traditionally used in vector-borne disease mapping. Covariates that were statistically significant to the age-corrected infection prevalence within each country were identified using a total-sets analysis based on a generalised linear regression model and were implemented in the bestglm package in R^19^, ^20^ separately for each country. Precipitation, temperature suitability index, enhanced vegetation index, and urbanisation were the set of parsimoniously selected covariates whose relations with *Pf*PR_2–10_ were explored (appendix).

Empirical data and spatially matched covariates were then used within a Bayesian hierarchical space–time model, implemented through an adapted stochastic partial differential equations (SPDE) approach with integrated nested Laplace approximations for inference,^21^, ^22^, ^23^ to produce continuous maps of *Pf*PR_2–10_ for 2000 and 2010 (appendix). In the SPDE approach, the overall hierarchical space–time binomial model of the parasite prevalence was represented as the realisation of a spatial–temporal process of the *Pf*PR_2–10_ at the community location and survey date, selected covariates at sampled locations, the coefficient vector, and the measurement error defined by the Gaussian white noise. The realisation of state process, or the unobserved level of *Pf*PR_2–10_, is defined by a Gaussian random field that changes temporally as a second-order autoregressive function. The space–time covariance matrix informs the spatial range and temporal lag of the prediction model for each country such that observations have decreasing effects on the predictions at a given location the more distal in space and time they are to that location. Outside of the spatial and temporal range, the autocorrelation of the data becomes almost null. Consequently, we used data from the period 1980–2012 to make predictions for the years 2000 and 2010 to harness the strength of the entire data while minimising the effect on predictions of spatially and temporally distant data. In the SPDE model, the Gaussian random field with its covariance function was represented as a Gaussian Markov random field. A non-stationary model was achieved by modifying the SPDE to obtain the Gaussian Markov random field with a defined dependence structure that is different from the stationary Mátern covariance. The local nature of differential operators was used to allow for local specification of the range and variance parameters.

This model was applied to produce continuous predictions of *Pf*PR_2–10_ at 1 × 1 km spatial resolutions for the year 2000 and 2010, which were generated by country. Data were generated for 49 malaria-endemic countries and territories in Africa. Zanzibar and Pemba and Bioko and Annobón were analysed separately from mainland Tanzania and Equatorial Guinea, respectively, because of their unique history of malaria control. For countries made of several islands (Cape Verde, Mayotte, Comoros, and São Tomé and Príncipe), data were analysed separately for each island.

### Populations at risk of varying stability and endemic classes and population-adjusted infection prevalence

The continuous *Pf*PR_2–10_ maps were classified into adapted traditional endemicity classes.^10^ We classified areas as those with predicted *Pf*PR_2–10_ less than 1% (low stable endemic control), which represents a pre-elimination transitional state;^14^ 1% to less than 5% (hypoendemic 1); 5–10% (hypoendemic 2); greater than 10% to 50% (mesoendemic); greater than 50% to 75% (hyperendemic); and greater than 75% (holoendemic). In addition to the malaria-free areas and the limits of unstable transmission for 2000 and 2010, there were eight malaria risks classes.

Population distribution and disease risks are both heterogeneous in space and time; therefore, estimates of population counts must be resolved to the highest possible levels of spatial detail to allow congruence with mapped disease risks. Modelling techniques for the spatial reallocation of populations within census units have been developed in an attempt to overcome the difficulties caused by input census data of low spatial resolutions (appendix).^24^ We used this gridded population surface for populations at risk in Africa in 2000 and 2010 by endemicity class. Within the stable limits of transmission, we computed population-adjusted *Pf*PR_2–10_ (PA*Pf*PR_2–10_) for each country by first multiplying the *Pf*PR_2–10_ in each 1 × 1 km area with the corresponding population at the same spatial resolution to compute the number of people who are likely to be positive for *P falciparum* by pixel by year. This surface was then used to extract the aggregate number of people positive for *P falciparum* and the total population in 2000 and 2010 per country to compute the mean PA*Pf*PR_2–10_ for each year.

### Model uncertainty and validation statistics

Using a spatially and temporally declustered algorithm, 10% of the *Pf*PR_2–10_ data was randomly held out per country. Model accuracy was estimated by computing the linear correlation, mean prediction error, and mean absolute prediction error of the observations and predictions to the holdout dataset. Additionally, maps of the number of SDs from the posterior mean *Pf*PR_2–10_ were generated for each 1 × 1 km grid location and were used to compute the percentage of population with one, two, or greater than two SDs of the mean *Pf*PR_2–10_.

### Role of the funding source

The sponsor of the study had no role in study design, data collection, data analysis, data interpretation, or writing of the report. AMN and RWS had full access to all the data in the study and had final responsibility for the decision to submit for publication.

## Results

**Figure 1:**
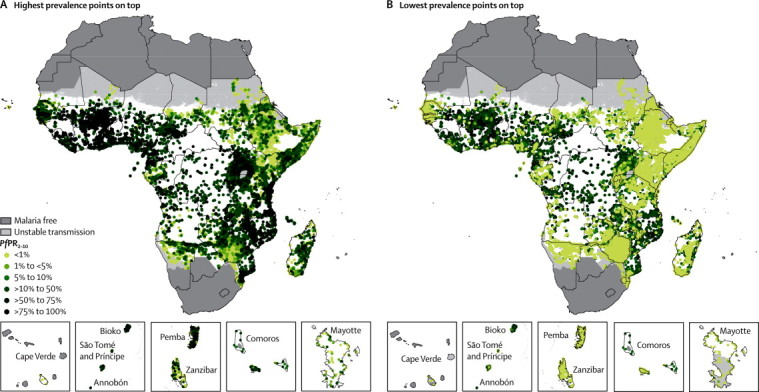
Distribution of 26 746 community *Plasmodium falciparum* parasite rate space–time survey datapoints in Africa for the period 1980–2012 (A) Highest prevalence points on top and (B) lowest prevalence points on top. The dark grey, light grey, and white areas are malaria free and the limits of unstable and stable transmission, respectively, for the year 2000 in Africa. *Pf*PR_2–10_=community *Plasmodium falciparum* parasite rate standardised to the age group 2–10 years.

The data search strategy identified 28 483 temporally, spatially, or both temporally and spatially unique *Pf*PR datapoints from surveys undertaken between January, 1980, and December, 2012, in 49 malaria-endemic territories in Africa (47 countries and the two pairs of islands of Zanzibar and Pemba and Bioko and Annobón). After a series of exclusions (appendix), the final model dataset included 26 746 space–time survey datapoints at 21 341 unique locations covering 3 575 418 person-observations. Figures 1A and 1B show the spatial distribution of these data with highest and lowest *Pf*PR_2–10_ values on top, respectively.

**Figure 2:**
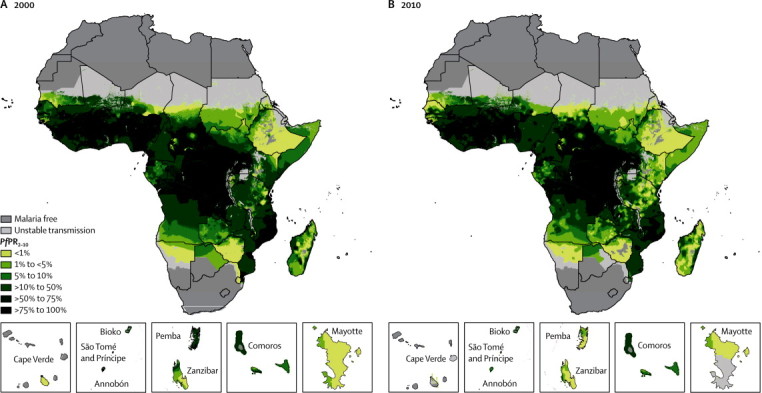
Predicted 1 × 1 km spatial resolution *Plasmodium falciparum* parasite rate endemicity class maps of Africa Prevalence in (A) 2000 and (B) 2010. The dark grey, light grey, and white areas are malaria free and the limits of unstable and stable transmission, respectively, for each year. *Pf*PR_2–10_ predictions were made to areas within the stable limits of transmission. *Pf*PR_2–10_=community *Plasmodium falciparum* parasite rate standardised to the age group 2–10 years.

**Figure 3:**
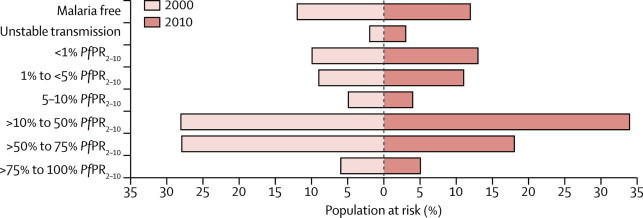
Percentage of population by *Plasmodium falciparum* parasite rate malaria endemicity class in endemic countries in Africa for the years 2000 and 2010 These estimates do not include Burundi, Central African Republic, Congo, Mauritania, and Niger, for which we did not have sufficient data to predict change. *Pf*PR_2–10_=community *Plasmodium falciparum* parasite rate standardised to the age group 2–10 years.

**Figure 4:**
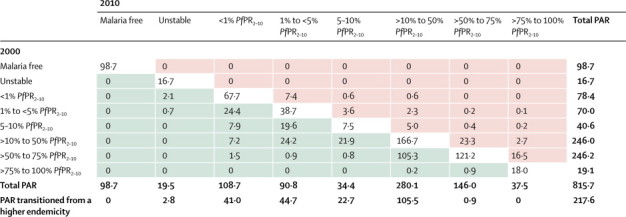
The 2010 population (millions) in malaria-endemic countries in Africa by their *Plasmodium falciparum* parasite rate endemicity class in 2000 and 2010 The green shaded cells show the number of people (millions) in 2010 who lived in areas where malaria endemicity had reduced by at least one level from that of 2000. The pink shaded areas are those where endemicity had increased by at least one level from that of 2000. These estimates do not include Burundi, Central African Republic, Congo, Mauritania, and Niger, for which we did not have sufficient data to predict change. PAR=populations at risk. *Pf*PR_2–10_=community *Plasmodium falciparum* parasite rate standardised to the age group 2–10 years.

**Figure 5:**
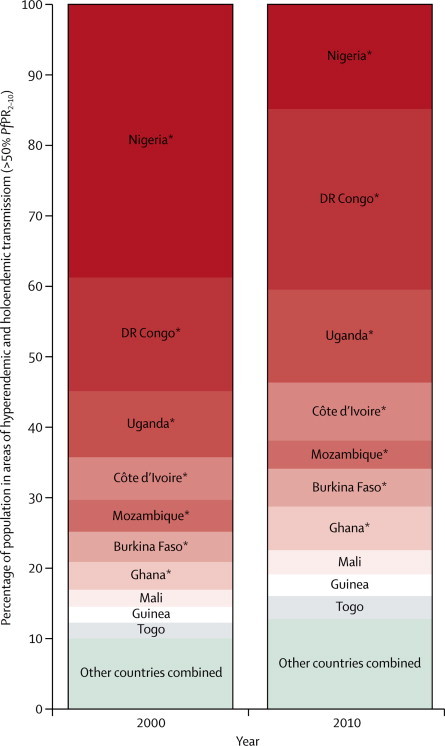
Bar charts of the population at risk in areas of hyperendemic and holoendemic transmission (>50% *Plasmodium falciparum* parasite rate) in 2000 and 2010 The ten countries shown here contribute to 87·1% of all populations living in hyperendemic and holoendemic areas in Africa in 2010. *Countries that are part of the ten countries in the WHO Malaria Situation Room.^25^*Pf*PR_2–10_=community *Plasmodium falciparum* parasite rate standardised to the age group 2–10 years.

Figures 2A and 2B show the predicted *Pf*PR_2–10_ endemicity class maps at 1 × 1 km resolution for 2000 and 2010. Data were insufficient to make reliable predictions between the two timepoints for five countries (Burundi, Central African Republic, Congo, Mauritania, and Niger), which were excluded in subsequent analyses of change. From 2000 to 2010, the percentage of the population within the stable limits of transmission living under hyperendemic (>50% to 75% *Pf*PR_2–10_) and holoendemic (>75% *Pf*PR_2–10_) transmission declined from 218·6 million (34·4%) of 635·7 million to 183·5 million (22·5%) of 815·7 million across the 44 countries combined (figure 3 and table). Populations living in areas of mesoendemic transmission (>10% to 50% *Pf*PR_2–10_) increased from 178·6 million (28·1%) in 2000 to 280·1 million (34·3%) in 2010. This increase was attributable mainly to populations transitioning from hyperendemic and holoendemic transmission (figure 4). The number of people living in areas where transmission was unstable or less than 1% *Pf*PR_2–10_ increased from 78·2 million (12·3%) in 2000 to 128·2 million (15·7%) in 2010. Within the rest of the hypoendemic belt of 1–10% *Pf*PR_2–10_, population increased from 84·7 million (13·3%) in 2000 to 125·2 million (15·3%) in 2010. The extent of the malaria-free areas changed marginally due to parts of central Zimbabwe being declared free of transmission by 2010. Most of the increase in population in malaria-free areas between the two prediction years was therefore attributable to natural population growth. Overall, the predicted change in transmission intensity translated into 217·6 million people (26·7%) in malaria-endemic countries in Africa living in a lower endemicity class in 2010 than in 2000 (figure 4). Only ten countries (Guinea, Togo, Mali, Mozambique, Burkina Faso, Ghana, Côte d'Ivoire, Uganda, Nigeria, and DR Congo) contributed 87·1% of the population (159·9 million of 183·5 million) living in areas of hyperendemic or holoendemic transmission in 2010 (figure 5).

**Table:** Population at risk (millions) by malaria endemicity class in 2000 and 2010 in malaria-endemic countries in Africa

	**2000 (n=635·7)**	**2010 (n=815·7)**
Malaria free	75·5 (11·9%)	98·7 (12·1%)
Unstable transmission	13·3 (2·1%)	19·5 (2·4%)
<1% *Pf*PR_2–10_	64·9 (10·2%)	108·7 (13·3%)
1% to <5% *Pf*PR_2–10_	53·5 (8·4%)	90·8 (11·1%)
5 to 10% *Pf*PR_2–10_	31·2 (4·9%)	34·4 (4·2%)
>10% to 50% *Pf*PR_2–10_	178·6 (28·1%)	280·1 (34·3%)
>50% to 75% *Pf*PR_2–10_	179·4 (28·2%)	146·0 (17·9%)
>75% to 100% *Pf*PR_2–10_	39·2 (6·2%)	37·5 (4·6%)

Data are number (%). *Pf*PR_2–10_=community *Plasmodium falciparum* parasite rate standardised to the age group 2–10 years.

**Figure 6:**
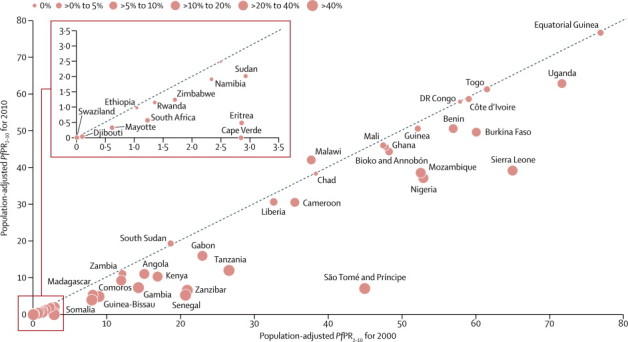
Bubble scatter plot comparing estimates of national mean *Plasmodium falciparum* parasite rate within the limits of stable transmission by country in 2000 and 2010 The size of the bubble dot shows the relative percentage change in national mean PA*Pf*PR_2–10_ between 2000 and 2010. Countries below the 45 degrees line are those where national mean PA*Pf*PR_2–10_ has declined whereas those above the line are those where it has risen. Points on the line are those with no change. These estimates do not include Burundi, Central African Republic, Congo, Mauritania, and Niger, for which we did not have sufficient data to predict change. The inset shows change in those countries in which national mean PA*Pf*PR_2–10_ in 2000 was less than 5%. Estimates for Cape Verde were those of Santiago Island only because the other Islands were already of unstable transmission by 2000.

A percentage reduction in national mean PA*Pf*PR_2–10_ of less than 1% to over 40% within the limits of stable transmission was estimated in 40 of 44 countries and territories where predictions could be made between 2000 and 2010 (figure 6). There was a percentage rise of mean PA*Pf*PR_2–10_ of 11% in Malawi and 4% in South Sudan, whereas DR Congo and Chad remained unchanged. In 2000, only Swaziland, Djibouti, and Mayotte had a mean PA*Pf*PR_2–10_ of less than 1%, a threshold of low stable endemic control. By 2010, these three countries were joined by Cape Verde (Santiago), Eritrea, South Africa, and Ethiopia. Of the 11 countries that were, based on national mean PA*Pf*PR_2–10_, classified as hyperendemic in 2000, four (Burkina Faso, Mozambique, Niger, and Sierra Leone) had achieved more than 10% change in infection prevalence such that by 2010 their national mean PA*Pf*PR_2–10_ were suggestive of mesoendemic transmission.

Estimates of *Pf*PR_2–10_ were associated with varying degrees of uncertainty, as shown by the number of SDs to the posterior mean and the magnitude of the linear correlations, mean prediction error, and the mean absolute prediction error (see appendix for model validation statistics by country and maps of number of SDs to the posterior mean *Pf*PR_2–10_). For most countries, most areas were predicted to within one SD of the posterior mean *Pf*PR_2–10_ for both 2000 and 2010. However, in parts of the Sahelian countries and Côte d'Ivoire, predictions were within two SDs of the posterior mean *Pf*PR_2–10_ and between two and four in most of Central African Republic and Niger. Excluding Central African Republic, Mauritania, and Congo, where data were too few to model any year, mean prediction error was greater than 5% *Pf*PR_2–10_ in only Benin and Togo, whereas the mean absolute prediction error was greater than 10% *Pf*PR_2–10_ in Benin, DR Congo, Guinea Bissau, and Togo.

## Discussion

We have assembled the largest geocoded repository of malaria infection prevalence data for Africa as part of an 8-year data search. We used these data to predict in space (1 × 1 km resolution) and time (2000 and 2010) the intensity of *P falciparum* transmission across malaria-endemic countries and territories. Our analysis estimates substantial reductions in *P falciparum* transmission intensity from 2000 to 2010 (panel). The population living in areas of highest transmission intensity (hyperendemic and holoendemic) decreased from 218·6 million in 2000 to 183·5 million in 2010, constituting a 16·1% change. 40 countries were predicted to have had a reduction in mean PA*Pf*PR_2–10_, and by 2010, 217·6 million people (26·7% of the population) in the malaria-endemic countries of Africa lived in areas where endemicity had reduced by at least one endemic class from that of 2000. In several areas that were of moderate-to-low transmission in 2000 there were large declines in transmission even though these areas remained within the same range of endemicity by 2010. Several countries are now at levels of transmission that probably warrant redefinition of their strategies for control under low transmission intensity or pre-elimination.

Panel **Research in context**

**Systematic review**

The period 2000–10 marks a decade of unprecedented investment in malaria control in Africa by the Roll Back Malaria Partnership. Attempts at tracking the changing burden of malaria during this period in Africa have focused on modelled estimations of clinical and fatal outcomes because of weak health information and civil registration systems and low parasitological confirmation of reported cases.^4^, ^5^, ^6^, ^7^, ^8^, ^9^ However, the modelled estimates of clinical and fatal outcomes rely on limited active case detection and verbal autopsy data. A more robust measure to track changing malaria risk is whether an individual sampled through random community surveys has evidence of infection after a blood examination. This metric has been used for over 100 years in Africa. Available descriptions of parasite prevalence in Africa predict the contemporary intensity of *Plasmodium falciparum*^15^, ^16^ and cannot be used to estimate changing risk. Herein, we have assembled the largest geocoded repository of *P falciparum* infection prevalence data for malaria-endemic countries in Africa from various published and unpublished sources as part of an 8-year data search to describe the changing *P falciparum* transmission intensity and population at risk in 2000 and 2010.

**Interpretation**

Substantial reductions in malaria transmission have been achieved in endemic countries in Africa over the period 2000–10, but 57% of the population in 2010 continued to live in areas where transmission was greater than 10% *Pf*PR_2–10_. Global funding for malaria control in Africa must remain a priority. This study provides information relevant to the improved estimation of the burden of malaria, the targeting of control interventions, and assessment of the determinants of changing *P falciparum* transmission intensity in Africa.

Despite these impressive reductions in the intensity of malaria transmission, 57% of people in Africa live in areas where risks remain mesoendemic or higher, with 87·1% of people in the two highest endemicity classes living in just ten countries. Of these, three (Guinea, Mali, and Togo) are not part of the ten countries that are the focus of the WHO Malaria Situation Room.^25^ The high population growth rates in Africa, which has resulted in almost 200 million additional people living in malaria-endemic regions of the continent in 2010 compared with 2000, has reduced some of the proportional gains in reduction of transmission.

In addition to providing a robust approach to quantification of the changing transmission of malaria in Africa, our work has several other important applications. First, the complex associations between the frequency of parasite exposure from birth, as measured by transmission intensity including the parasite rate, and the likelihood of disease outcomes have been used previously to derive estimates of the numbers of clinical and fatal cases at continental and global scales.^4^, ^5^, ^6^, ^7^, ^8^ These models were based on limited clinical and verbal autopsy data, but need a more robust understanding of changing parasite exposure to improve the precision in trends in disease burden estimates.

Second, why the intensity of *P falciparum* has changed so dramatically in some areas and seems to be intractable in others over the past decade is a fundamental question for future investment in malaria control in Africa. In this study, our aim was not to assess the determinants of the changing transmission intensity—these are probably a combination of the effect of malaria interventions, the increasing levels of urbanisation and human settlement patterns, improved socioeconomic status, climate, and other short-term and long-term factors. To quantify the effect of the investment in malaria control over the past decade and to forecast future changes in malaria burden needs an extensive assembly of data to understand this complex array of factors. However, the information in this study provides a unique opportunity to unravel this complex combination of factors within and between countries in Africa. This work is being pursued in partnership with several countries in the region.

Third, using only the 2010 map to target the future needs of universal vector control is inappropriate. Areas of less than 1% *Pf*PR_2–10_ at present might have been of higher transmission intensity a decade earlier. An inability to reach and sustain universal coverage in these areas could result in a rebound. Conversely, areas that were less than 1% *Pf*PR_2–10_ in 2000 and continued to be in 2010 need a different approach to the control and elimination of malaria. There is also a growing consensus from mathematical models that baseline transmission intensity is a crucial determinant of the effect size of vector control.^12^, ^13^, ^26^ Areas that have historically been described as intense transmission areas, for example greater than 50% *Pf*PR_2–10_, need more than one vector control strategy to reduce transmission to less than 1% *Pf*PR_2–10_. From our analysis the areas of largest reduction in transmission intensity were those where transmission in 2000 was less than 50% *Pf*PR_2–10_, for example, Senegal, The Gambia, and areas within the east Africa region (Kenya, Rwanda, and Tanzania). Therefore, the design of malaria control strategies depends upon an understanding of transmission intensity both before and after intervention.

In this study, we focused on national estimates of change in transmission intensity and populations at risk. However, we recognise that for within-country applications, detailed (ie, high spatial resolution) information that represents the heterogeneity of malaria is a valuable instrument for routine malaria programme planning. We have presented 1 × 1 km predictions of endemicity and continuous *Pf*PR_2–10_. Ongoing work with regional countries is aimed at providing estimates of risk and populations at risk at subnational administrative unit levels that are relevant to decision making for malaria. This work will support subnational resource allocation, budgetary gap analysis, malaria programme reviews, and updates of national malaria strategies.

The data and analysis presented herein is a marked improvement on previous approaches to modelling of malaria parasite transmission intensity in Africa.^15^, ^16^ We have substantially improved the temporal and spatial distribution of the data; increased the precision in geocoding of survey locations; and reduced potential uncertainties by increasing minimum sample size thresholds for data inclusion, implementing analysis by country, and substantially minimising the cloud contamination in enhanced vegetation index surfaces (appendix). However, we acknowledge several limitations to our approach that are important to the interpretation of the results and their future applications. There were countries that had limited data for 2000 or 2010, or both. These countries were Central African Republic, Congo, and Mauritania for both years; Chad and DR Congo for 2000; and Burundi and Niger for 2010. We excluded Central African Republic, Congo, Mauritania, Burundi, and Niger from the analysis of changing transmission. These five countries account for only 4·2% of the estimated 848 million people in malaria-endemic countries in Africa in 2010.^24^ For DR Congo and Chad, we assumed that no change in malaria transmission had occurred between the two timepoints because of the low coverage of malaria control interventions by 2010.^4^ In the prediction of *Pf*PR_2–10_, we used only urbanisation and long-term average temperature suitability index, enhanced vegetation index, and precipitation to improve model precision. These covariates do not necessarily capture the secular patterns in climate and urbanisation and their effect on transmission. Use of time-varying covariates in large-scale mapping exercises such as this is computationally intensive and would have made modelling of pixel-level risk difficult even with access to super-computing. We also did not include malaria control intervention coverage in the prediction of *Pf*PR_2–10_ because these data are not available at the temporal and spatial scales of either the climate covariates or the parasite prevalence data. We discuss the potential effects on our analysis of the variations in parasite detection methods and minimum sample size thresholds we have chosen in the appendix.

In conclusion, we provide an empirical measure of transmission intensity across malaria-endemic countries in Africa in 2000, soon after the launch of the Roll Back Malaria partnership, and compare this with that of 2010. These two timepoints are important benchmarks for global health and development goals and represent a decade of unprecedented investment in malaria control.^3^, ^4^ Our analysis suggests substantial reductions in malaria transmission intensity during this decade. We show that, by 2010, more than a quarter of Africa's population lived in areas where malaria endemicity had declined by an order of magnitude of one or more class. In most countries there has been a reduction in transmission and fewer people now live in areas of the highest transmission intensity. These reductions probably contributed to progress towards the disease burden targets in the Global Malaria Action Plan^27^ and the Millennium Development Goals.^28^ However, the epidemiological transition has not been universal and 57% of Africa's population continue to live in areas of moderate-to-high transmission intensity. In a period of global economic recession, these results emphasise the need for continued support for malaria control, not only to sustain the gains that have been made, but also to accelerate the reduction in transmission intensity where it still remains high. If investments in malaria are not sustained, hundreds of millions of Africans run the risk of rebound transmission, with catastrophic consequences.
