# Supplementary material for: The changing risk of Plasmodium falciparum malaria infection in Africa: 2000–10: a spatial and temporal analysis of transmission intensity
Source: Lancet. 2014 May 17;383(9930):1739–47. doi: 10.1016/S0140-6736(13)62566-0 (PMC4030588; doi:10.1016/S0140-6736(13)62566-0)
Supplement: Supplementary appendix [file mmc1.pdf]

THE LANCET

Supplementary appendix

This appendix formed part of the original submission and has been peer reviewed. We post it as supplied by the authors.

Supplement to: Noor AM, Kinyoki DK, Mundia CW, et al. The changing risk of *Plasmodium falciparum* malaria infection in Africa: 2000–10: a spatial and temporal analysis of transmission intensity. *Lancet* 2014; published online Feb 20. http://dx.doi.org/10.1016/S0140-6736(13)62566-0.

dWebappendix for Original Research Article

The changing risk of *Plasmodium falciparum* malaria infection in Africa: 2000 to 2010

Abdisalan M Noor, Damaris K Kinyoki, Clara W Mundia, Caroline W Kabaria, Jonesmus W Mutua, Victor A Alegana, Ibrahim Socé Fall, Robert W Snow

Background: Following a decade of the Roll Back Malaria Partnership and unprecedented investment in malaria control, we have examined for the first time, the change in malaria transmission intensity during the period 2000-2010 in Africa.

Methods: We assembled a geocoded and age-corrected community *Plasmodium falciparum* parasite rate (*Pf*PR$_{2\text{-}10}$) database from across 49 endemic countries and territories in Africa from surveys undertaken since 1980. The data were used within a Bayesian space-time geostatistical framework to predict *Pf*PR$_{2\text{-}10}$ in 2000 and 2010 at a 1 × 1 km spatial resolution. Population distribution maps at the same spatial resolution were used to compute populations at risk by endemicity class and estimate population adjusted *Pf*PR$_{2\text{-}10}$ (PA*Pf*PR$_{2\text{-}10}$) for each of the 44 countries where predictions were possible for each year.

Findings: Between 2000 and 2010 the population in hyperendemic (>50% to 75% *Pf*PR$_{2\text{-}10}$) or holoendemic (>75% *Pf*PR$_{2\text{-}10}$) areas had reduced from 218.6 million (35%) to 183.5 (23%) in 44 malaria endemic countries. 280 million(34%) people lived in areas of mesoendemic transmission (>10% to 50% *Pf*PR$_{2\text{-}10}$) in 2010 compared to 178.6 million (28%) in 2000. Population in areas of unstable or very low transmission (<5% *Pf*PR$_{2\text{-}10}$) had increased from 131.7 million people in 2000 to 219 million in 2010. An estimated 217.6 million people, or 27% of the 2010 population, lived in areas where transmission had reduced by at least one *Pf*PR$_{2\text{-}10}$ endemicity class. Forty countries showed a reduction in national mean PA*Pf*PR$_{2\text{-}10}$. Only ten countries contributed 87.1% of the population living in areas of hyperendemic or holoendemic transmission in 2010.

Interpretation: Significant reductions in malaria transmission have been achieved in endemic countries in Africa over the period 2000-2010. However, more than two-thirds of the population in 2010 continue to live in areas where transmission remains moderate to intense and global support to sustain and accelerate the reduction of transmission must remain a priority.

Funding: This work was funded by the Wellcome Trust. The funder played no role in design of the study or interpretation of the results.

Table of Contents

W1 Defining the limits of *Plasmodium falciparum* transmission and population in 2000 and 2010 3

W1.1 Defining transmission stability 3

W1.2 Medical intelligence to define the absence and stability of *P. falciparum* transmission 3

W1.2.1 Countries deemed P. falciparum free 3
W1.2.2 The changing extent and stability of P. falciparum transmission across Africa's islands 4
W1.2.3 The changing extent and stability of P. falciparum transmission in southern Africa 4
W1.3 The transmission limiting effects of temperature and aridity 5

W1.3.1 Temperature 5
W1.3.2 Aridity 5
W1.4 Population distribution 2000 and 2010 8

W2 Parasite prevalence data search, assembly, pre-processing and summaries 10

W2.1 Background 10

W2.2 Data search strategy 10

W2.2.1 Electronic data searches: 10
W2.2.2 Unpublished archived survey reports: 10
W2.2.3 Unpublished community survey data post-2005 11
W2.2.4 Search completeness 11
W2.3 Data abstraction 12

W2.4 Data geo-coding 12

W2.5. Database fidelity checks and exclusions 13

W2.6 Data summaries 14

W2.7 Age standardization 15

W3 Covariate processing/selection and model procedures 17

W3.1 The problem of over-fitting in variable selection 17

W3.2 Assembling plausible covariates of *Pf*$PR_{2\text{-}10}$ 17

W3.4 Model-based geostatistics (MBG) 21

W3.5 Model specification 22

W3.5.1 Constructing a suitable MESH 24
W3.5.2 Model outputs 24
W3.5.3 Model Validation 27
W3.6 Rationale for not selecting data from borders with neighbouring countries 29

W4 References 30

W1 Defining the limits of *Plasmodium falciparum* transmission and population in 2000 and 2010

W1.1 Defining transmission stability

The stability of malaria transmission is defined quantitatively by the average number of feeds that a mosquito takes on man during its life[1]. The stability index, however, demands detailed entomological data that are rarely available. Qualitatively, stable malaria refers to situations that are relatively insensitive to natural and man-made changes. Unstable malaria includes areas very sensitive to climatic aberrations and very amenable to elimination. Critical to the planning of elimination during the Global Malaria Eradication Programme, fifty years ago, was a quantitative description of risk for planning control and monitoring progress. During the preparatory phase, large-scale parasite prevalence surveys were undertaken to examine feasibility of elimination. It was suggested that when infection prevalence became very low, national programmes should invest in combinations of passive, active and mass-blood survey surveillance of new infections, expressed as an annual parasite incidence (API) per 1000 people resident in a reporting administrative area [2-4].

These epidemiological definitions have recently been reviewed to identify transitional points from sustained malaria control or a pathway toward elimination[5-7], and adapted here. We have used the reported absence of transmission or absence of clinical events to define areas free of *P. falciparum* risk in combination with a temperature mask to exclude the possibility of parasite transmission from vectors to man. We used case-incidence data, where these exist, to define unstable malaria < 1 *P. falciparum* case per 10,000 population per annum and stable malaria ≥ 1 *P. falciparum* case per 10,000 population per annum. In addition, we have used a biological constraint on the ability to support stable transmission and classified all extremely arid areas as those more likely to support unstable transmission.

W1.2 Medical intelligence to define the absence and stability of *P. falciparum* transmission

An assembly of reports, case-incidence data and communications with national malaria control programme managers was undertaken to define the extent of active *P. falciparum* transmission[8]. Information has been digitized using administrative boundary units in ArcGIS (version 10.1, ESRI Inc. USA).

W1.2.1 Countries deemed P. falciparum free

Plasmodium falciparum transmission probably reached its natural extent in Africa around 1900[9] and how these historical limits of transmission have changed over the last 100 years is described in detail elsewhere[8]. The Kingdom of Lesotho; the Islands of the Seychelles archipelago (Tromelin, Cargados Carajos and Agalega); Rodrigues, Saint Brandon and Chagos in the Mascarene archipelago; and the island of Saint Helena, in the Atlantic Ocean, are African territories that have never supported malaria transmission[10]. The Western Sahara was reported in 1955 by the Spanish governing authorities to be completely free from malaria transmission[11] and the neighbouring Northern areas of Mauritania, Adrar, Inchiri, Dakhlet-Nouadhibou and Tiris Zemmour, north of 20°N are also regarded as malaria free[12].

By 2000, almost all of the North African territories were *P. falciparum* free. *P. falciparum* free status was achieved in Libya in 1973, the Kingdom of Morocco in 1974, Tunisia by 1979, and the extent of falciparum transmission dramatically reduced by 1978 in Algeria with the exception of small area on the border with Mali, where *P. falciparum* transmission remains uncertain by 2010[13]. The last possible case from a residual focus of transmission in Fayoum Governorate in the United Arab Republic of Egypt was in 1998. We treat both Algeria and Egypt as *P. falciparum* free in 2000.

In Reunion, the elimination strategy was launched in 1949[14] and eventually led to the elimination of both *P. vivax* and *P. falciparum* by 1973[15]. Mauritius was a highly endemic island at the turn of the last century[16,17], the first elimination campaign was launched in 1948. The WHO certified Mauritius malaria-free in 1973. *P. falciparum* transmission has yet to re-establish itself on the island despite a resurgence of *P. vivax* transmission in 1975.

Between 1910 and 1973 transmission of malaria was essentially absent from the Republic of Djibouti, where after it is thought that both parasites and vectors arrived by train from neighbouring Ethiopia leading to several epidemics in the early 1980s and sustained stable transmission in populated areas until 2000. By 2010, however, case incidence had declined significantly and the majority of Djibouti is now best described as unstable[18].

W1.2.2 The changing extent and stability of P. falciparum transmission across Africa's islands
The Republic of Cape Verde launched a malaria elimination campaign in 1948, starting on the island of Sal and extended to other Islands throughout the 1950s. The campaign was successful and malaria was felt to have been eliminated in Sal (1950), Sao Vicente (1954), Boavista and Maio (1962) and Santiago (1968)[19,20]. With the exception of Santiago, no autochthonous cases were detected for many years on any of the islands since they were declared malaria free. However, malaria transmission was re-established on the island of Santiago in 1973. In 1979, a further national elimination programme was launched and the focus was on Santiago and the entire archipelago was returned to zero incidence between 1983 and 1986[21]. In 1995–1996 an epidemic occurred in St. Catarina district on Santiago[22]. However, in 2000 and 2010 locally acquired case incidence was below 1 per 10,000 people on Santiago (mainly Santa Catarina and Santa Cruz) and therefore regarded as unstable. On Boavista malaria had been absent since 1962, including 2000, however four possible autochthonous cases were detected in 2003, 10 cases in 2009 and three in 2010 rendering Boa Vista an area of unstable transmission in 2010.

In Mayotte (Mahore and Pamanzi islands), located within the Comorian Archipelago *P. falciparum* transmission has been intense and stable for many years[23]. Through integrated vector and disease control in the 1980s, parasite prevalence declined to less than 0.3% and only eight clinical cases were reported by 1988[24]. However, resurgent waves of transmission continued through the early 1990s. By 2000 malaria transmission had returned to a stable state across the islands[25]. By 2010 case incidence had declined again and using detailed case data it is possible to delineate areas of unstable and stable case incidence across the islands of Mayotte[26,27].

The two volcanic islands that make up the Democratic Republic of São Tomé and Príncipe have never been free of malaria and have maintained stable transmission despite attempts at elimination[28- 33]. There were two concerted elimination efforts on the island of Zanzibar prior to the launch of RBM, both without success[34,35]. Despite a significant reduction in parasite transmission on the island since 2000, stable endemic transmission continues[36]. Its sister island of Pemba has always supported stable transmission. The three islands that form the Federal Islamic Republic of Comoros (Grand Comore, Anjouan, and Moheli) have had high *P. falciprum* disease rates since the 1920s[37-39] and despite significant control efforts from 2007, prevalence and partial clinical data suggest that the three islands remain at stable, endemic transmission[40].

W1.2.3 The changing extent and stability of P. falciparum transmission in southern Africa
A careful assembly of historical evidence of risk in South Africa pre-1940 suggests that malaria was absent from large parts of the western region and areas bordering the southern reaches of Namibia and Botswana[41-43]. Sustained efforts to eliminate malaria since the Second World War in the Transvaal and KwaZulu-Natal provinces rendered increasing areas unstable and reduced the spatial extent of risk[43-46]. Stable endemic risks in South Africa were constrained in 2000 to areas located along the Kruger national park, borders with Zimbabwe in the Limpopo, Mpumalanga provinces and the two northern districts of Ingwavuma and Ubombo in KwaZulu-Natal province. By 2010, case incidence in South Africa had dropped dramatically and case incidence by district has been used to delineate unstable and stable areas risks in Limpopo and Mpumalanga and unstable risks in Ingwavuma, KwaZulu-Natal Province.

In 1950, a national malaria reconnaissance was undertaken across Namibia; the most southerly areas from Grootfontein and Franzfontein to the Orange River were regarded as free of malaria transmission or very focal pockets of occasional transmission[47]. Similarly today the southern provinces of Karas and Hardap are regarded as malaria-free[48,49]. Districts located immediately north of Windhoek had some clinical cases between 2000 and 2010 and these have been mapped by health administration unit to reflect areas where annual incidence was < 1 per 10,000 population per annum[50] and regarded as unstable transmission by the Ministry of Health and Social Services[51]. All other districts supported stable transmission in 2000 and 2010.

Malaria risk in the Republic of Botswana is constrained by latitude and the Kalahari Desert (that makes up 70% of the country's land mass). In 1958, 98% of all malaria cases were reported from two districts, Ngamiland and Chobe, in the North in areas surrounding the Okavango and Chobe swamps fed by the Zambezi River[52]. During a national parasitological survey in 1961–1962, no sampled infants were found to harbour infections in Tsha, Loda, Gaborone, Kanye, Moduchi, Tuli and Ghanzi areas[53]. Botswana reported no cases in South-East, North & South Kgalagadi, Southern, East & West Kweneng, Kgatleng, Good Hope, Lobatse and Gaborone health districts in 1999 and are treated as malaria free in 2000[42]. Over the following years the health districts of Seleb Phikwe, Francistown, Serowe Palapye, Ghanzi, North East, Mahalapye and Tutume reported very few cases and classified as unstable in 2010[54].

In Zimbabwe, since the 1920s, autochthonous transmission of malaria in Harare and Bulawayo has not been reported[55-57]. The various efforts to control and eradicate malaria over the years probably led to constrained areas of unstable transmission since the 1950s; by 1979, the central districts were regarded as malaria free. However, malaria case incidence rose significantly across the country from the mid-1980s to a point in 2000 when the country had returned to a stable endemic state. The investment in control since 2000 has rendered 12 districts in central highlands malaria free (Goromonzi, Seke, Marondera, Hwedza, Chikomba, Chirumanzu, Gutu, Gweru, Shurugwi, ZviShavane, Chivi and Umguza) that now form part of a consolidation phase of elimination[58].

In the Kingdom of Swaziland, following the Second World War, successive efforts at malaria control led to an infant malaria parasite rate of 0% by 1956[59] and IRS was stopped in 1959. By 1970, it is stated that the only cases were those imported from outside the country[60] and malaria operations were drastically scaled down. During the early 1980s a resurgence of malaria risk was witnessed, briefly abated, and rising again through to a peak in the late 1990s. By 2000 the disease burden was constrained to Lubombo and Hhohho region[60]; the highveld has continued to be malaria free[61]. Following a renewed malaria control effort, malaria case incidence has begun to decline from 2000[62], and for the three consecutive years 2006–2008, incidence was below 1 per 10,000 population to a point in 2010 where transmission is unstable risk across the regions of Lubombo and Hhohho, with one stable endemic district of Mhlangatane, and the remaining areas the Kingdom ostensibly malaria free.

Figure W1.1a shows the limits of stable and unstable transmission based upon medical intelligence in 2000 across 49 endemic countries and territories.

W1.3 The transmission limiting effects of temperature and aridity

W1.3.1 Temperature: Temperature plays a key role in determining the transmission of human malaria[63]. To provide a plausible mask to eliminate the possibility of transmission across Africa, we have used a recently developed temperature suitability index (TSI)[64]. The TSI model uses a biological framework based on the survival of vectors and the fluctuating monthly ambient temperature effects on the duration of sporogony that must be completed within the lifetime of a single generation of Anophelines. This was used to generate at each 1 x 1 km pixel, periods of an average year when a vector's lifespan would exceed the time required for sporogony, and hence when transmission was not precluded by temperature. If this time exceeded the maximum feasible vector lifespan, then the cohort was deemed unable to support transmission and the area classified as being at zero risk[64]. Applying this temperature mask highlights the highland areas and mountains of East Africa, the southern mountains of Tanzania, the mountains at the junction of Democratic Republic of Congo, Rwanda and Burundi, the highlands in Ethiopia, Mount Cameroun, the Shimbiris mountains in Somaliland, the Nyika Plateau in Malawi, Mount Nyangani in Eastern Zimbabwe, and the volcanic mountain of Karthala on Grand Comores (Figure W1.1 b).

W1.3.2 Aridity: Arid conditions effect anopheline development and survival[65]. Limited surface water reduces the availability of sites suitable for oviposition and reduces the survival of vectors at all stages of their development through the process of desiccation[66]. We have defined extreme aridity using the enhanced vegetation index (EVI) derived from Moderate Resolution Imaging Spectrometer (MODIS) satellite imagery[67]. Monthly EVI datasets archived over 11 years were averaged to a synoptic year and used to classify into areas likely to support transmission, defined by an EVI of greater than 0.1 for any two consecutive months and areas without two or more consecutive months of an EVI > 0.1 as unable to support transmission[68, 69]. This aridity mask identifies small foci of risk across the Sahara that are likely to support stable transmission because of their proximity to oases and seasonal rivers, while retaining a plausible mask of almost zero transmission across the Sahara, the arid, barren areas of Eritrea (including the Danakil depression), Djibouti and Somalia and the deserts in southern Africa including the Sossusvlei and Skeleton Coast. This mask we have used conservatively as representing unstable transmission, rather than malaria free, as transmission might be possible in the presence of man-made water bodies such as dams or underground water storage (Figure W1.1d).

Figure W1.1 a) the limits of stable and unstable transmission based upon medical intelligence in 2000 across 50 endemic countries; b) the combined effects of medical intelligence and temperature on the limits of stable and unstable transmission in 2000; c) the combined effects of medical intelligence, temperature and aridity on the limits of stable and unstable transmission in 2000; d) the combined effects of medical intelligence, temperature and aridity on the limits of stable and unstable transmission in 2010. Dark grey = malaria free; light grey = unstable transmission; white = stable transmission.

a)

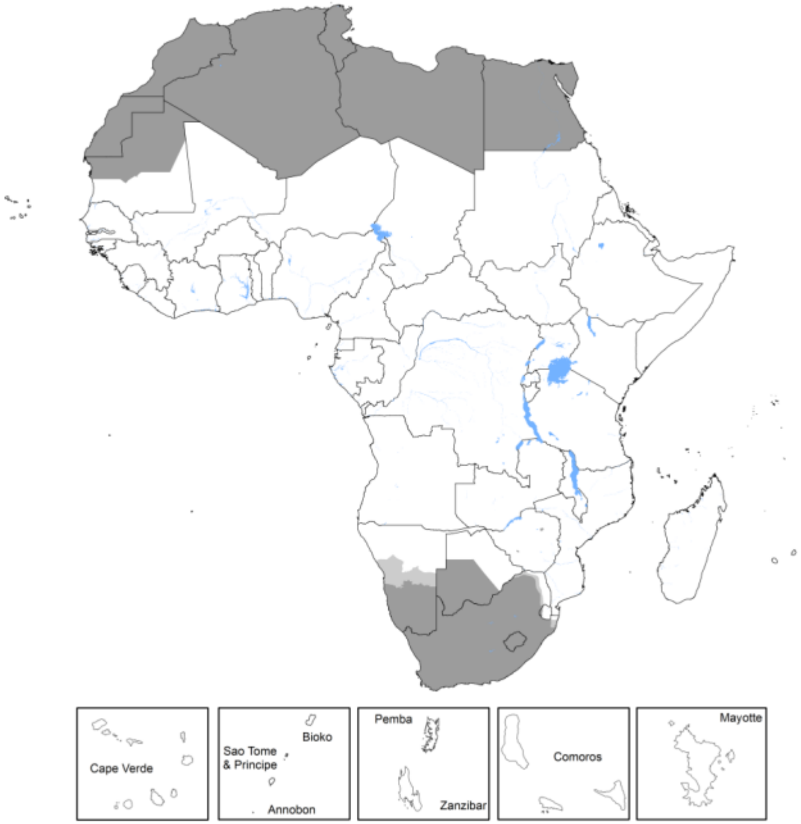

b)

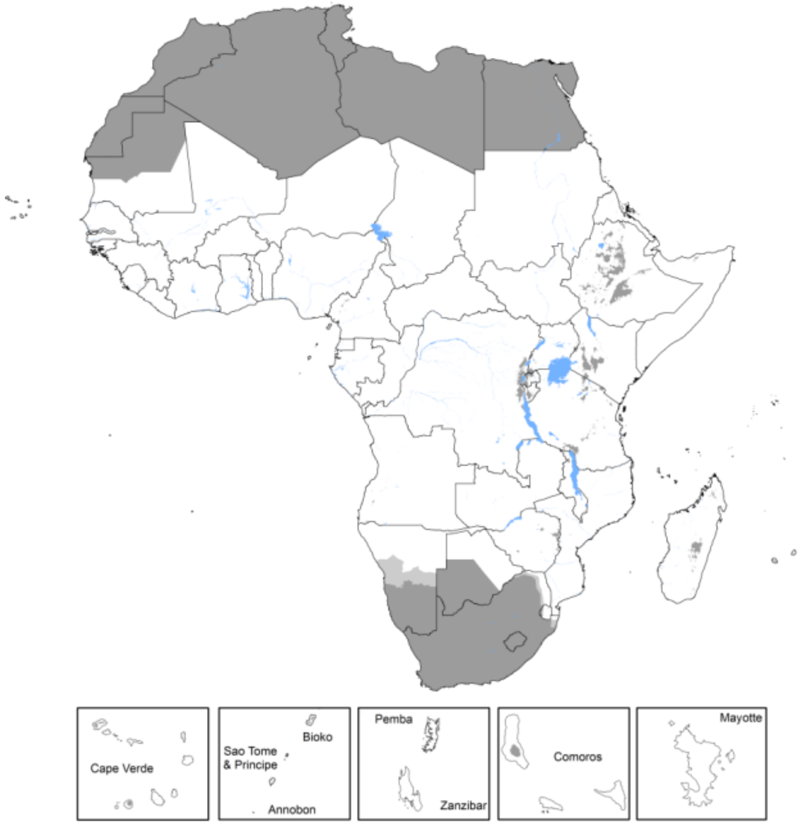

c)

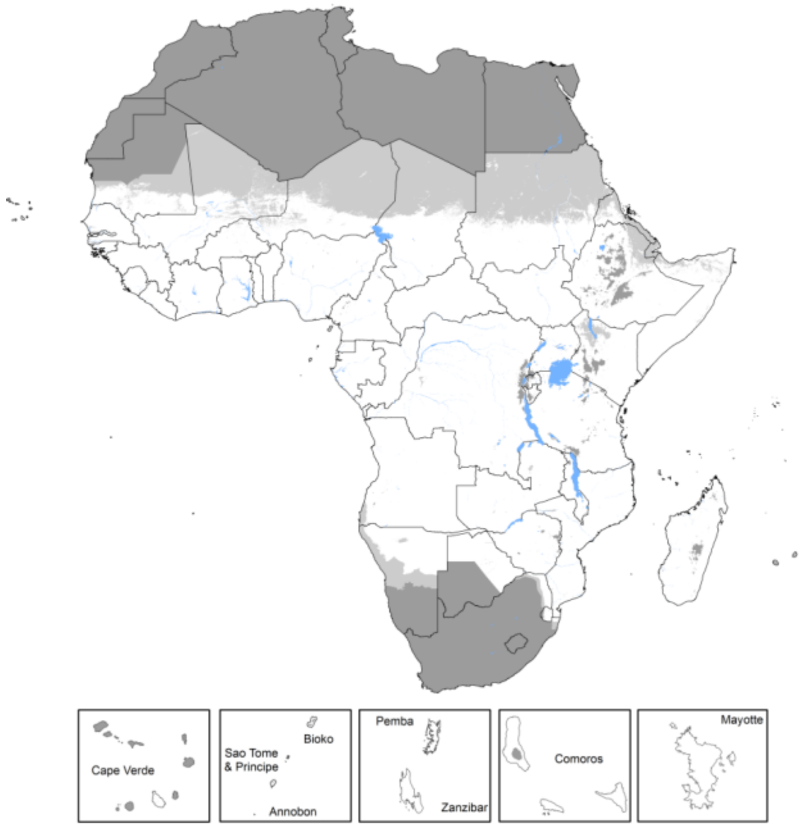
Cape Verde
Sao Tome
& Principe
Bioko
Annobon
Pemba
Zanzibar
Comoros
Mayotte

d)

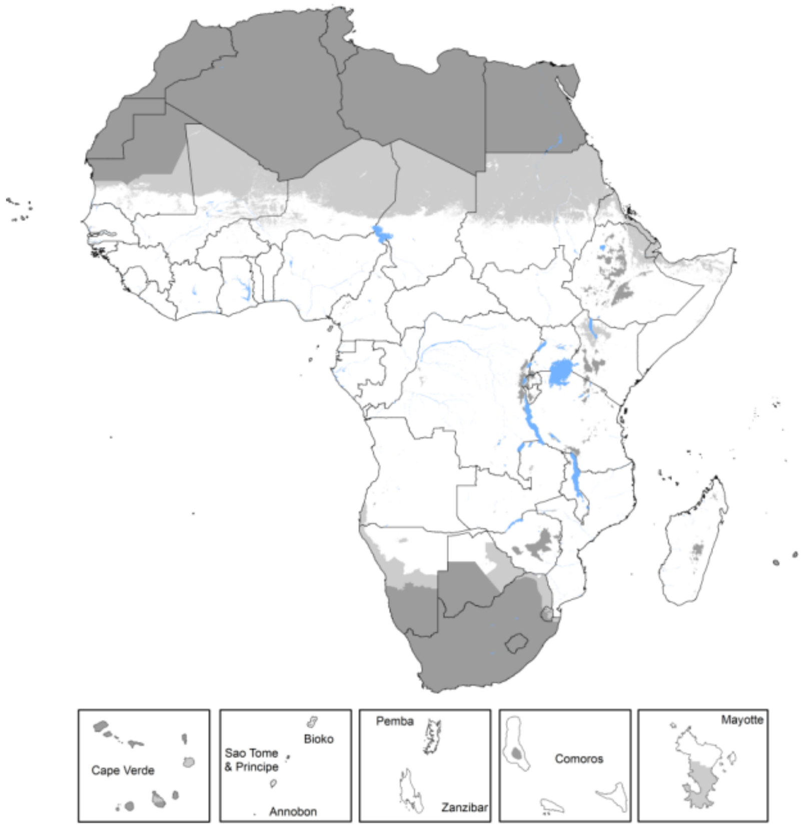
Cape Verde
Sao Tome
& Principe
Bioko
Annobon
Pemba
Zanzibar
Comoros
Mayotte

W1.4 Population distribution 2000 and 2010

Where disease risks are heterogeneous in space, population distributions and counts should be resolved to higher levels of spatial detail than large regional estimates. For many low income countries of the World, however, spatially detailed, contemporary census data do not exist. This is especially true for much of Africa, where currently available census data are often over a decade old, and at administrative boundary levels just below national-level. Modelling techniques for the spatial reallocation of populations within census units have been developed in an attempt to (a) disaggregate population count data to a finer spatial detail and (b) convert population count data from irregular administrative units to regular raster layers[70-72]. In brief, a dasymetric modelling technique[73] was used to redistribute census population counts within administrative unit boundaries based on land cover data sets derived from satellite imagery to produce a gridded dataset of population distribution (counts) at 0.1 x 0.1 km resolutions. Human population census data, official population size estimates and corresponding administrative unit boundaries at the highest level available from the most recent available censuses were acquired for each African country. Typical regional per-land cover class population densities were estimated from African countries for which very fine resolution population data were available, following approaches outlined elsewhere[71]. These typical population densities were then applied as weightings to redistribute census counts according to the land cover and to map human population distributions at a finer spatial resolution. The population datasets were projected to 2000 and 2010 using national rural and urban growth rates estimated by the UN Population Division[74].

The urban extents produced by the Global Rural-Urban Mapping Project (GRUMP)[75] were used to distinguish between urban and rural areas. Finally, datasets were adjusted to ensure that national population totals matched those reported by the UN[76]. Population distribution was re-sampled to 5 x 5km grids. Figure S1.2 shows the spatial distribution of population across stable and unstable malaria endemic regions of sub-Saharan Africa in 2000.

We have used actual census data from the islands of Cape Verde (2010), São Tomé and Príncipe (2012), Zanzibar (2002), Comoros (2003) and Mayotte (2007) projected using UN census growth rates to 2000 and 2010 on the islands and districts within the islands at malaria risk in each of these years.

Figure W1.2 Predicted population density distribution within margins of *P. falciparum* transmission in 2000 based on medical intelligence (FigureW1.1a). ☐ **<1 person per km^2; ☐ < 10 people per km^2; ☐ 10-49 people per km^2; ☐ 50 - 99people per km^2; ☐ 100 – 499 people per km^2; ☐ 500 - 1000 people per km^2; ☐ > 1000 people per km^2**

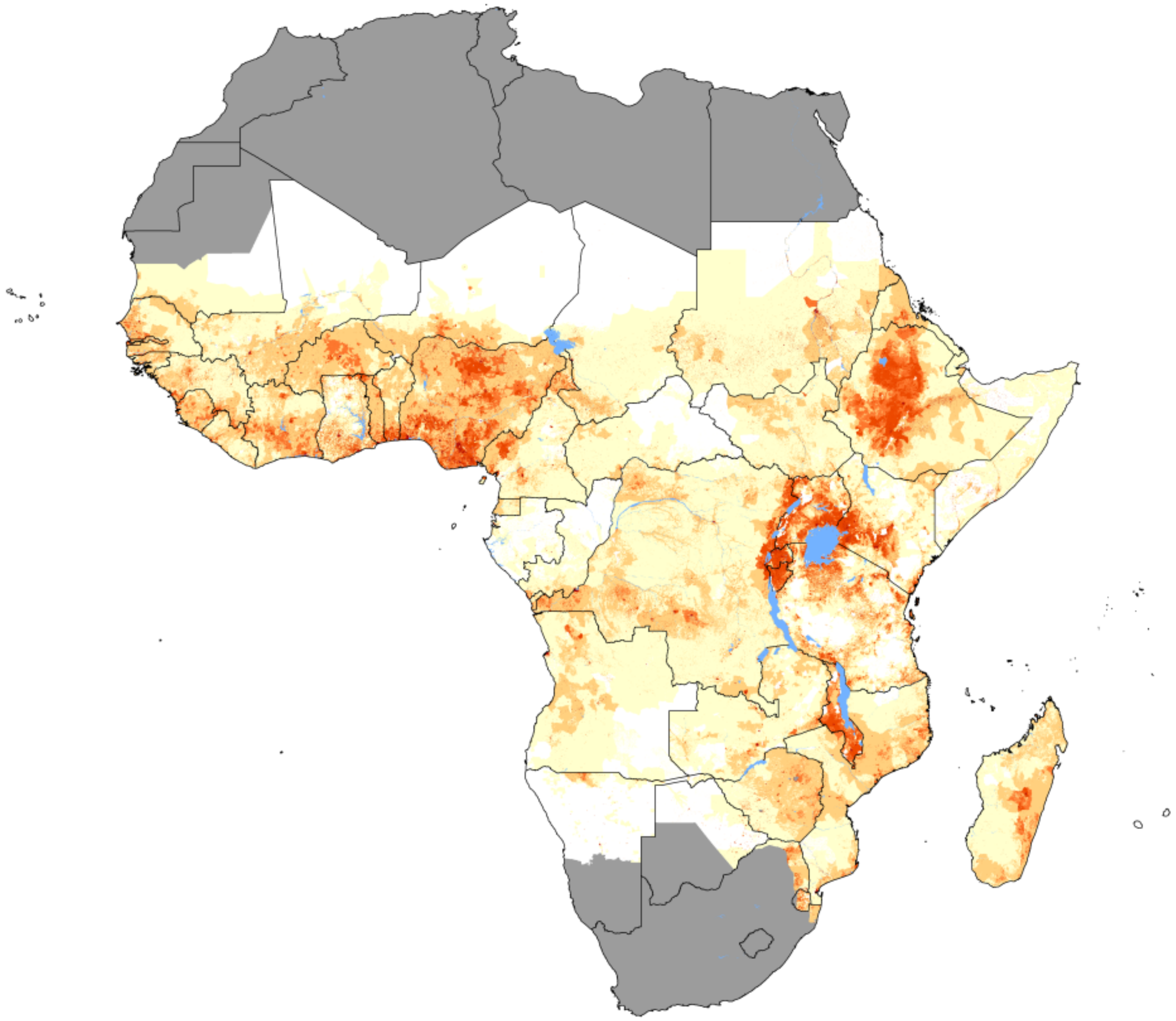

W2 Parasite prevalence data search, assembly, pre-processing and summaries

W2.1 Background

There are a variety of measures of the intensity of malaria transmission derived from field investigations of human populations or vectors. The most ubiquitous measure, used for over 100 years in Africa, is the parasite rate - the proportion of individuals on a single cross-sectional survey among an entire or sampled community who have evidence of a peripheral blood stage malaria infection. This data are often expressed as infection prevalence among children aged 2-10 years ($Pf\text{PR}_{2\text{-}10}$) and used since the 1950s to define categories of endemic risk designed to guide and monitor progress toward malaria elimination targets[77-79].

W2.2 Data search strategy

W2.2.1 Electronic data searches: Online electronic databases were used as one means of identifying peer-reviewed, published data on malaria infection prevalence including: PubMed[80]; the Armed Forces Pest Management Board – Literature Retrieval System[81]; the World Health Organization Library Database[82]; and the Institute de Recherché pour le Development on-line digital library service[83]. Regional journals, most notably the large number of national medical, public health and parasitological journals, were not identified readily from the above sources but titles and abstracts were available on African Journals Online (AJOL)[84]. In all digital electronic database searches for published work, the free text keywords "*malaria*" and "*country-name*" were used. We avoided using specialised Medical Subject Headings (MeSH) terms in digital archive searches to ensure as wide as possible search inclusion. Database searches were undertaken at least once per year from 2005 and the last complete digital library search was undertaken in March 2013. Searches were supplemented through routine weekly notifications from Malaria World[85], the Roll Back Malaria news alert service[86], the Environmental Health at USAID malaria bulletin[87] and Santé Tropicale for Francophone country national and regional journals including Medecine D'Afrique Noire[88].

Titles and abstracts were used to identify possible parasite cross-sectional survey data undertaken since January 1980 in a variety of forms: either as community surveys, school surveys, other parasite screening methods or intervention trials. We also investigated studies of the prevalence of conditions associated with malaria when presented as part of investigations of anaemia, haemoglobinopathies, blood transfusion or nutritional status to identify coincidental reporting of malaria prevalence. In addition, it was common practice during early antimalarial drug sensitivity protocols to screen community members or school attendees to recruit infected individuals into post-treatment follow-up surveys, often data from the survey sites selected present the numbers screened and positive. Surveys of febrile populations or those attending clinics were excluded.

Publications with titles or abstracts suggestive of possible parasite data were either downloaded from journal archives where these have been made Open Access (OA) or sourced from HINARI[89]. If publications were not available OA from HINARI we visited UK library archives at the London School of Hygiene and Tropical Medicine, the Liverpool School of Tropical Medicine, the Bodleian library at the University of Oxford, Wellcome Trust and British library or the Library at the Institute Pasteur, Paris to obtain copies. References not found following these searches were requested using world catalogue searches through the Oxford libraries at a per-page cost. All publications from which data were extracted were cross-referenced using the bibliographies for additional sources that may have been missed or that may correspond to unpublished or 'grey' literature, not controlled by commercial publishers.

W2.2.2 Unpublished archived survey reports: A wealth of unpublished research and survey reports are available at archives in various European Tropical Medicine Institutes. We undertook manual searches of archives at the Institute of Tropical Medicine, Antwerp; Institute Pasteur, Paris; Department of History of Medicine, Sapienza - Università di Roma, Rome; ArchivioItaliano Di Scienze Mediche Coloniali, Rome; Instituto Higiene Medicina Tropical, Lisbon; UK national archives and the World Health Organization (WHO) libraries in Geneva, Cairo and Brazzaville.

We also visited national libraries of Ministries of Health at Nairobi, Kisumu, Eldoret, Mombasa and Meru (Kenya), Entebbe (Uganda), Sennar, Khartoum and Kassala (Sudan), Thies (Senegal), Amani (Tanzania), Tzaneen (South Africa) and the personal archives of the ex-director of Tzaneen Malaria Research centre (covering South Africa, Namibia and Botswana). At each European or African archive survey data were located and wherever possible,

electronic copies (Adobe PDF) were made of all original survey reports. Finally we used the digital archive of survey data developed under the Mapping Malaria Risk in Africa (MARA/ARMA) initiative, established by one of us (RWS) in collaboration with colleagues from South Africa and other regional centres in 1996[90,91].

W2.2.3 Unpublished community survey data post-2005: As part of the Roll Back Malaria (RBM) monitoring and evaluation national, household surveys were resurrected as a means to monitor country-level progress[92,93]. These surveys were initially embedded in the Demographic and Health Surveys (DHS) as a malaria module and were largely focussed on intervention coverage measures until 2005 when it was agreed to include malaria infection prevalence into survey protocols. These two-stage random cluster selected national surveys included combinations of methods parasite detection in different age groups depending on the national survey. Where these surveys were co-managed by the US based MEASURE-ICF programme, data from these national surveys have been made available on-line[94]: Angola (2006 and 2011), Burkina Faso (2010), Cameroon (2011), Liberia (2009 and 2011), Madagascar (2011), Malawi (2012), Mali (2010), Nigeria (2010), Rwanda (2007 and 2010), Senegal (2008 and 2010), Tanzania (2007-8 and 2011-12), Uganda (2009) and Zanzibar (2011-12).

Adapted forms of these survey approaches have been used by national malaria control programmes (NMCPs) as standalone Malaria Indicator Surveys (MIS). Data from these national surveys have been generously provided by Ministries of Health and survey partners covering: Chad (2011), Democratic Republic of Congo (DRC) (2009), Djibouti (2009), Equatorial Guinea (2007 and 2010), Eritrea (2002), Ethiopia (2006, 2007 and 2011), The Gambia (2008 and 2010), Ghana (2011), Guinea Bissau (2008), Kenya (2007 and 2010; national malaria schools survey 2009-2010), Malawi (2000 and 2009), Mozambique (2002 and 2007), Namibia (2009), Somalia (2005), South Sudan (2009), Republic of Sudan (2005 and 2009), Swaziland (2010), Zambia (2006, 2008, 2010 and 2012) and Zanzibar (2007 and 2010).

For other possible unpublished, site-specific data on malaria prevalence we reviewed web-sites and contacted Non-Governmental Organizations (NGO) working across Africa who may have undertaken health assessments that included parasitological investigations of communities where they worked. These included Médecins sans Frontières[95], MERLIN[96], MENTOR[97], the Carter Center[98], The Food Security and Nutrition Analysis Unit - Somalia (FSNAU)[99], Shape Consulting[100] and the Malaria Consortium[101]. Contacts through these agencies led to identification of data from sites in Guinea, Sierra Leone, Côte D'Ivoire, Tanzania, Liberia, Somalia, Kenya, Uganda, Nigeria, Ethiopia and South Sudan. Most notable has been the established collaboration with the FSNAU in Somalia and our Malaria Public Health Department in Kenya, where we have worked in partnership to include malaria parasitology into routine nutritional and food security surveys since 2007[102].

Tropical Medicine and malaria meeting abstract books were identified from as many sources as possible produced as part of national and international conferences and congresses. These were used to signal possible data that were followed up through correspondence with abstract authors. Our regional presence and connections to the wider African malaria research community has created an awareness of the purpose and ambition of malaria mapping research first started in 1996 under MARA[90]. This regional connectivity of research scientists was used to contact directly colleagues working on the epidemiology of malaria to seek disaggregated site-specific and often unpublished data. The willingness to share unpublished parasite survey data has been unprecedented, 651 individual scientists and public health specialists have contributed disaggregated information on published data, unpublished data or have assisted in the location of sampled communities across Africa, all are acknowledged at our webpage[103].

W2.2.4 Search completeness: Our data searches have not used systematic, traditional evidence review strategies. These would have missed many unpublished sources of information. Rather our strategy has used a cascaded, opportunistic approach. Authors of peer-reviewed papers were often asked about additional information within their paper and directions to other possible unpublished work in their geographic area or from their institution. Searches of European tropical Institute's libraries and archives focussed only on the major established tropical centres of those who had colonial ties to Africa. We have not visited every country in Africa to search for national Ministry of Health data nor had access to every national parasitology research conference proceedings, it is notable that our archived data is greatest for Kenya, Tanzania, Uganda, Senegal and Namibia where we have had access and searched archived national data.

National sample surveys undertaken in recent years but not made available for inclusion before July 31st 2013 include surveys in Benin (2011), Burundi (2012), Côte D'Ivoire (2012), Equatorial Guinea (2011), Guinea (2012),

Mali (2012), Sierra Leone (2011) and Zimbabwe (2009 and 2012). None of these datasets have been released by the MEASURE-ICF program or national collaborating partners. Similarly we have not been able to access known national and sub-national surveys of nutritional status undertaken in Ghana (1999) and Kenya (2010) that included investigations of malaria infection prevalence. We have tried to identify as many post-graduate research theses as we could through on-line resources in the UK, however obtaining digital copies of older theses was not possible and thus remain a possible source of unpublished data not archived by us. More importantly are the many post-graduate theses undertaken by students of the faculties of parasitology, public health and medicine at the many universities across Africa that have not been adequately searched.

W2.3 Data abstraction

The minimum required data fields for each record were: description of the study area (name, administrative divisions and geographical coordinates, if available), the dates of start and end of the survey (month and year) and information about blood examination (number of individuals tested, number positive for *Plasmodium* infections by species), the methods used to detect infection (microscopy, Rapid Diagnostic Tests (RDTs), Polymerase Chain Reaction (PCR) or combinations) and the lowest and highest age in the surveyed population. Given its ubiquity as a means for malaria diagnosis, the preferred parasite detection method was microscopy. No differentiation was made between light and fluorescent microscopy. The quality of slide reading[104,105], variations in sensitivity/specificity between RDTs[106] or the ability of field teams to reliably read RDTs[107,108]and selection of primers for PCR[109] all influence descriptions of prevalence and will have intrinsic variance between surveys included in the database. RDTs have been shown to yield higher false positive rates than microscopy[106] but seem to stratify both the lowest (<1% parasite rate) and highest (> 50% parasite rate) more accurately compared to routine microscopy[105]. An analysis of a large collection of community parasite rate data have shown that there was minimal difference in estimates of overall mean *P. falciparum* prevalence in matched paired analysis of community survey data that used microscopy and RDT for parasite examination[110].

For data derived from randomized controlled intervention trials, data were only selected when described for baseline, pre-intervention and subsequent follow-up cross-sectional surveys among control populations. When cohorts of individuals were surveyed repeatedly in time we endeavoured to include only the first survey and subsequent surveys if these were separated by at least five months from the initial survey to avoid dependence between observations based on treatment of preceding infected individuals. If it was not possible to disaggregate repeat surveys these were finally excluded from the analysis. Where age was not specified in the report for each survey but stated that the entire village or primary school children examined we assumed age ranges to be 0-99 years or 5-14 years respectively. Occasionally, reports presented the total numbers of people examined across a number of villages and only the percentage positive per village; here we assumed the denominator per village to be equivalent to the total examined divided by the total number of villages. It was possible to establish the year of every survey; however, the month of survey was occasionally not possible to define from the survey report. Here we used descriptions of "wet" and "dry" season, first or second school term or other information to make an approximation of the month of survey and included a record of this assumption. Some survey results were reported as an aggregate in space (e.g. a single *Pf*PR for a group of villages) or time (e.g. a mean *Pf*PR estimated from four different surveys conducted over time). In such cases, we either sought additional reports of the same surveys with higher spatial or temporal resolution. Where this was not possible and where clusters of villages exceeded 5 km^2 we excluded the record from the analysis (see below). Where additional information to provide unique time, village specific data was necessary we contacted authors to provide any missing information.

W2.4 Data geo-coding

Data geo-coding, defining a decimal longitude and latitude for each survey location, was a particularly demanding task. According to their spatial representation, data were classified as individual villages, communities or schools or a collection of communities within a definable area, corresponding to an area within a 5 km grid or approximately 0.04 decimal degrees at the equator. Where possible we aimed to retain disaggregated village, "point" level data rather than data across a "wide-area". Where data were reported across communities that exceeded at 5 km grid we regarded these as too low a spatial resolution, with significant possible variation within the polygon of information to be excluded within the modeling phase. In practice this was a difficult criterion to audit as most survey reports did not provide enough detail on the size of the area surveyed. More recent use of Global Positioning Systems (GPS) during survey work does enable a re-aggregation of household survey data with greater precision and useful in

maintaining 5 km grid criteria while combining clusters of small sample sizes in space. To position each survey location where GPS coordinates were not available in space we used a variety of digital resources, amongst which the most useful were Microsoft Encarta Encyclopedia (Microsoft Inc., 2004) and Google Earth (Google Inc., 2009). Other sources of digital place name archives routinely used included GEOnet Names Server of the National Geospatial-Intelligence Agency, USA[111]; Falling Rain Genomics' Global Gazetteer[112]; and Alexandria Digital Library prepared by University of California, USA[113]. Across Africa a number of national digital, GPS confirmed, place-name gazetteers exist for populated places, health facilities or schools. These are increasing in number, precision and coverage. These were obtained on request from national census bureau's, ministries of education and health and NGO partners and proved to be valuable locating communities in Kenya, Uganda, Burkina Faso, The Gambia, Mauritania, Zambia, Mozambique, Madagascar, Somalia, Malawi, Ghana, Burkina Faso, Niger, Namibia, South Africa, Tanzania and Zanzibar. Although standard nomenclatures and unique naming strategies are attempted in digital gazetteers[114], these are difficult to achieve at national levels where spellings change between authors, overtime and where the same place names are replicated across a country. As such, during the data extraction, each data point was recorded with as much geographic information from the source as possible and this was used during the geo-positioning, for example checking the geo-coding placed the survey location in the administrative units described in the report or corresponded to other details in the report on distance to rivers or towns when displayed on Google Earth. While in theory GPS coordinates should represent an unambiguous spatial location, these required careful re-checking to ensure that the survey location matched the GPS coordinates. As routine we therefore rechecked all GPS data from all sources using place names and/or Google Earth to ensure coordinates were located on communities.

All coordinates were subject to a final check using second level administrative boundary Global Administrative Units Layers (GAUL) spatial database developed and revised in 2008 by Food and Agriculture Organization (FAO) of the United Nations[115]. The spatial selection tool in ArcGIS 10.1 (ESRI, USA) was used to verify points along the coastline were within land area as defined by GAUL 2008. The Global lakes and Wetlands (GLWD) database developed by the World Wildlife Fund[116] was used to ensure inland points were within defined land area. Here we aimed to identify survey coordinates that fell slightly off the coastline, located on the river or in incorrect administrative units, every anomaly was re-checked and re-positioned using small shifts in combination with Google Earth.

W2.5. Database fidelity checks and exclusions

Data checks: The entire database was first checked with a series of simple range-check constraint queries to identify potential errors that could have occurred during data entry. These queries assessed all data fields relevant to modelling for missing or inconsistent information. The final objective was to check for any duplicates introduced during the iterative data assembly process. Pairs of survey sites found within 1 km or within five months at the same location were identified. These may have entered erroneously into the data assembly where multiple reports reviewed describing similar data. These were listed, checked and duplicates removed.

Data exclusions: The search strategy identified 28,483 time-survey locations where malaria infection prevalence had been recorded between 1st January 1980 and 31st December 2012. This final data series was then subjected to various exclusion rules as defined below.

Location details: Despite repeated efforts and multiple on-line digital gazetteers, national resources and personal communications we were unable to identify with sufficient precision the geo-coordinates for 540 (1.9%) survey data points at 500 unique locations.

Polygon and multiple repeat surveys: We were unable to obtain higher spatial resolution data from 23 survey reports from 13 locations that described prevalence across areas exceeding 5 km^2 in Sudan (13), Comoros (3), Tanzania (2), Bioko Island of Equatorial Guinea (1), Gabon (1), Ghana (1) and Zambia (1). Sixteen unique locations were surveyed more than once and it was not possible from the original survey reports or attempts to communicate with investigators to separate out the first from subsequent surveys in Mali (10), Senegal (2), Sudan (2), Côte d'Ivoire (1) and Cameroon (1).

Ensuring sample precision: Sample size is inversely related to prevalence where, at low sample sizes, biases in prevalence estimates are introduced, dependent on the true prevalence of the population and translates into large

standard errors[117]. There is a critical threshold of between 10 and 20 individuals sampled below which the standard error increases exponentially in most surveys of parasitic infections and the curve starts to flatten at a sample size of about 50 and reaches an asymptote at about 100[118]. The sample size of individual survey samples is also important in the derivation of correlations with covariates of endemicity, in testing plausible associations between say rainfall and prevalence during covariate selection where small, imprecise samples can lead to over-fitting (Table W3.4). We aimed to combine communities in close proximity where any village had less than 15 people sampled and where communities were within 5 km of each other, sampled at exactly the same time by the same investigators. Using these criteria we were still unable to merge data from 1,191 (4.2%) time-space locations and these were excluded from the final analysis (see Table W2.1 for country distribution).

Rational inclusion of repeat annual Mass Blood Surveys: Several data series were the result of annual Mass Blood Surveys undertaken over decades within sentinel villages of defined spatial areas of The Republic of Sudan and Namibia. To improve the processing efficiency in these spatial areas we have thinned the data to represent data in five year intervals. Specifically, since 1999 in The Republic of Sudan two intensive surveillance areas have been subject to annual investigations of infection prevalence: Geizera[119] and the Gambiae Eradication project area in the north at Wadi Halfa[120]. Here we have reduced the data volume at each location to surveys undertaken nearest to the years 2000, 2001, 2005 and 2010 resulting in the exclusion of 903 survey points at Gezira and 344 survey points at Wadi Halfa. In Namibia, intensive indoor residual house spraying across the northern territories was accompanied by annual Mass Blood Surveys at sentinel villages between 1968 and 1992[49], here we retained data for periods that were 1980, 1985 and 1990 or the nearest year to these five-year intervals among villages sampled repeatedly across the interval, resulting in the exclusion of 662 survey data points.

Coastal and lake islands or malaria free locations: Off of Africa's coastline and the shores of its major inland lakes several small islands exist. These pose problems for approaches of matching environmental covariate grids (seeW3.3) as the sizes of the islands are often less than 5 km^2 and not captured by existing climate and ecology datasets. We have identified all off-shore, small islands from within the data set and have excluded from the analysis 304 (1%) data points where surveys were undertaken on these islands. These island exclusions included 137 survey data points from the islands of Bukooli, Bujumba, Bunya, Busiro, Buvuma, Kyamusua and Mukono parishes of Uganda located in Lake Victoria; 66 data points from Wasini, Lamu, Pate and Shella islands in the Indian Ocean and islands belonging to Suba District in Lake Victoria in Kenya; 34 survey data points from the Islands of Likoma in Lake Malawi; 26 survey points from the archipelago of Bolama/Bijagos islands in the Atlantic, off the coast of Guinea Bissau; seven survey data points on Lagos Island, Nigeria; five data points from Nosy Be island in the north of Madagascar; two survey data points from Lagunes area of Côte D'Ivoire; four data points located on islands in Lake Mweru, Zambia; one from the island of Fernan-vaz, Gabon; two from the island of Mafia, Tanzania; and one from Bol located in Lake Chad, Chad. In addition, 56 time-space survey locations were sampled in areas of South Africa (51), Harare, Zimbabwe (1), Kingdom of Swaziland (1) and Mauritania (3) that by 2000 were classified through medical intelligence (W1.2) as malaria free. These were removed from the final analysis data set.

W2.6 Data summaries

The eight year data search identified 26,746 temporally unique data points at 21,341 survey locations undertaken since January 1980 using the inclusion criteria described above (see Table W2.1 for country distribution). Every endemic country in Africa provided some data across this time period. The final prevalence database comprised of information on temporally separate survey locations sourced from peer-reviewed journals (4761, 17.8%), other archived reports including those of Ministries of Health (8995, 33.6%), conference abstracts (515, 1.9%), post-graduate theses (289, 1.1%), the MARA digital archive (79, 0.3%; unpublished data from Benin, Burkina Faso, Cameroun, Central African Republic, Chad, Congo, Côte D'Ivoire, Gabon, Mauritania and Togo between 1980 and 2004); and other sources that required the provision of unpublished data (12186, 45.6%). The latter representing the most important source of empirical data provided by researchers, ministries of health or NGOs identified initially from other sources, including reports and peer-reviewed publications or only through personal communication.

Survey data were located for time-space survey data points using GPS (11508, 43%), Encarta (2785, 10.4%), Google Earth (1596, 6%), GeoNames (689, 2.6%), other digital place names sources available at country-levels (4175, 15.6%) %), coordinates provided by individual scientists for which sources not certain (1156, 4.3%). Of the 26,746 time-space survey location data 18,539 (69.3%) used microscopy alone, 720 (2.7%) RDTs with microscopic

confirmation, 6812 (25.5%) RDTs alone, 298 (1.1%) RDTs with PCR confirmation, 329 (1.2%) PCR alone; and 48 (0.2%) microscopy with PCR confirmation.

W2.7 Age standardization

There was a large diversity in the age ranges of sampled populations between studies. To make any meaningful comparisons in time and space a single standardized age range is required. Correction to a standard age for *Plasmodium falciparum* is possible based on the observation and theory of infectious diseases where immunity is acquired following repeated exposure from birth. We have retained the classical age range of 2-10 years as this best describes the exposure to infection among semi-immune hosts at any given location and conforms to classifications established in the 1950s[77]. We have adapted catalytic conversion Muench models, first used in malaria by[121], into static equations in R-script that uses the lower and upper range of the sample and the overall prevalence to transform into a predicted estimate in children aged 2-10 years, $PfPR_{2\text{-}10}$[122].In brief, this algorithm computes the probability that an individual of a known age will be positive for *P. falciparum* infection as a function of the observed presence of infection, the age-dependent sensitivity of detecting infections and the age distribution of sampled populations[122].

Table W2.1: Description of data assembly by country 1980-2012. Time/or space location survey data excluded because of a) inability to find longitude and latitude and b) sample surveyed at location less than 15.

Country	Unable to locate	Small sample size	# 1980-2012	# 1980-2012 Spatially Unique
Angola	3	25	281	276
Benin	0	0	69	56
Bioko & Annobon[1]	0	2	106	43
Botswana	5	1	330	97
Burkina Faso	31	38	509	425
Burundi	1	1	190	149
Cameroon	4	91	365	339
Cape Verde	0	0	7	4
Central African Republic	0	0	13	10
Chad	0	6	244	242
Comoros	1	0	59	39
Congo	0	0	25	24
Côte d'Ivoire	0	1	252	227
Djibouti	0	0	140	138
DRC + Angola (Cabinda)[2]	0	1	411	398
Equatorial Guinea	0	10	49	30
Eritrea	40	1	347	344
Ethiopia	0	0	1380	1285
Gabon	0	2	195	187
Gambia	0	0	692	536
Ghana	44	119	341	328
Guinea	24	1	140	140
Guinea Bissau	3	3	151	143
Kenya	10	41	3794	2969
Liberia	0	18	281	163
Madagascar	6	11	842	654
Malawi	0	83	921	895
Mali	2	7	315	247
Mauritania	0	0	33	23
Mayotte[3]	0	0	141	58
Mozambique	11	123	873	619
Namibia	135	24	1320	833
Niger	1	0	58	52
Nigeria	12	29	653	613
Rwanda	1	14	742	732
São Tomé and Príncipe	0	0	71	35
Senegal	21	63	541	508
Sierra Leone	0	0	44	43
Somalia	91	35	1877	1458
South Africa	8	0	148	119
South Sudan	7	8	145	142
Sudan	10	52	1843	1055
Swaziland	0	5	183	176
Tanzania	26	289	2193	1447
Togo	0	2	231	153
Uganda	21	23	969	830
Zambia	0	40	939	873
Zanzibar & Pemba[4]	1	19	396	300
Zimbabwe	27	3	897	884
**Totals**	**546**	**1191**	**26746**	**21341**

Footnotes:

1. The islands of Bioko and Annobon form part of Equatorial Guinea but are 32 km from the coast and are analysed separately

2. Four survey points from Angola (South Central tile) sampled in the separated Cabinda territory have been included in the Central African tile owing to their proximity to Congo and the Democratic Republic of Congo; 3 points surveyed in 1985/6, one point surveyed in 2006. Two survey locations in 2011 in Cabinda were of small samples sizes and excluded.

3. Although Mayotte is an overseas department and region of France, under the UN regional grouping it is grouped with the Eastern African region with a Numerical Code of 175 (http://unstats.un.org/unsd/methods/m49/m49regin.htm)

4. The Islands of Zanzibar and Pemba form part of the United Republic of Tanzania but are represented here separately

W3 Covariate processing/selection and model procedures

W3.1 The problem of over-fitting in variable selection

In statistical modelling, a set of independent covariates of the main outcome measure is often used to improve the model fit and increase the precision of predicted estimates [123-126]. The inclusion of these covariates increase model complexity and, if not carefully selected, risk over-fitting (using up too many degrees of freedom), which occurs when more terms or covariates than is necessary are used in the model fitting process [123,125,126]. Over-fitting can lead to poor quality predictions because coefficients fitted to these covariates add random variations to subsequent predictions and make replication of findings difficult [123]. Where too many covariates are used, the model tends to produce highly fluctuating regression coefficients increasing the chances of large covariate coefficients and an overly optimistic fit, especially with small sample sizes[123]. This problem can be particularly pronounced when data assembled are from observational studies based on different study designs, sampling considerations and sample sizes which are then combined to describe a random process[125]. The considerations and procedures for the selection of covariates have therefore been described as one of the most important stages of the statistical modelling process[124,125,127].

The choice of these covariates should be underpinned by the principle of parsimony (few strong and easily interpretable covariates) and plausibility (a clearly understood mechanism by which the covariate influences the outcome)[123-125]. In disease mapping there must be a pre-determined aetiological explanation of the relationship of the disease and the covariate under consideration. Including in the prediction process covariates whose relationship with the outcome cannot be explained *apriori* may still lead to a 'good' model fit but this may largely be an artefact. To model the risks of *P. falciparum* malaria transmission in time and space, we have implemented a process of assembling a minimum, important set of covariates that have a biologically plausible relationship with malaria transmission and subjected them to a stringent selection process for inclusion into subsequent predictive models.

W3.2 Assembling plausible covariates of *Pf*$PR_{2\text{-}10}$

The determinants of malaria transmission are climatic (rainfall and temperature), ecological (potential breeding sites and urbanisation) and control interventions (anti-vector and ant-parasitic measures)[128,129]. These factors affect the development and survival of the *P. falciparum* parasite and the malaria-transmitting *Anopheles* vector thereby reducing the risks of infection. These covariates must be spatially and temporally matched with the observed data on transmission to account for their effect in time and space. However, they are rarely available at time points that correspond with the date of surveys. This is mainly because most of the covariate information is derived from long-term processed remotely sensed satellite imagery, which have become available at sufficiently high resolution only in the last decade [130] or modelled climatic data generated as synoptic estimates that do not represent a specific year[131]. Even where available, the inclusion of time-varying covariates, while statistically attractive, increases the complexity of MBG methods substantially to the extent that computations may be intractable where large datasets are involved. Regarding malaria control interventions, in addition to poor availability of space-time matching data, their inclusion creates an intrinsic circularity when analysing the role of intervention coverage against changing transmission, an area we hope to address in the future. For these reasons, the assembled covariates were long-term annual average raster grid representations of the climatic and environmental determinants of transmission and described in more detail below.

Temperature: Laboratory experiments have shown that high temperatures (>34 ^{o}C) lead to almost 100% larval mortality and at lower temperatures (<16°C) the larvae were unable to produce viable adults [132,133]. The mortality of the anopheles mosquitoes also increase sharply at ambient temperatures approaching 40 ^{o}C[134,135]. Temperatures of between 25°C and 30°C are considered optimum for *P. falciparum* sporogony[128].

It is on the basis of these biological relationships that we have assembled two temperature metrics in order to test their statistical relationships with *Pf*$PR_{2\text{-}10}$. These were: annual mean temperatures; and a biologically modeled temperature suitability index (TSI). The annual mean temperature surface was developed from monthly average temperature raster surfaces at 1 × 1 km resolution which were downloaded from the WorldClim website [136]. These surfaces were produced from global weather station temperature records gathered from a variety of sources for the period 1950-2000 and interpolated using a thin-plate smoothing spline algorithm, with altitude as a covariate, to produce a continuous global surface [131] (Figure W3.1a). TSI, as explained in W1.3.2, was developed as a quantitative measure of optimal *P. falciparum* sporozoite development at 1 × 1 km spatial resolution [64]. The TSI model uses a biological framework based on the survival of vectors and the fluctuating

monthly ambient temperature effects on the duration of sporogony that must be completed within the lifetime of a single generation of Anophelines. The TSI is constructed using long-term monthly temperature time series[131] and represented on a scale of increasing transmission suitability, from 0 (unsuitable) to 1 (most suitable) (Figure W3.1b).

Proxies of suitable conditions for larval development (precipitation and vegetation): Rainfall, combined with suitable ambient temperatures, provides potential breeding environments for *Anopheles* vectors while humidity is associated with vector longevity [137,138]. Normally, proxies of rainfall such as precipitation and vegetation are used in malaria risk predictions in Africa[131,125,130,139]. This is because actual rainfall data, typically collected from weather stations, are sparse throughout the continent[9].

Monthly mean precipitation raster surfaces at 1 × 1 km resolution were downloaded from the WorldClim website[136] and used as a proxy for rainfall compiled from weather stations over a similar period as the mean temperature surfaces[131]. These monthly surfaces were summed to generate a synoptic annual mean precipitation surface (Figure W3.1c). For vegetation, Fourier–processed enhanced vegetation index (EVI), derived from the Moderate-resolution Imaging Spectroradiometer (MODIS) sensor imagery and available at approximately 1 × 1 km spatial resolution[130] was used to develop an annual mean EVI surface. EVI is an index of intensity of photosynthetic activity and ranges from 0 (no vegetation) to 1 (complete vegetation) (Figure W3.1d). EVI, compared to the more commonly used Normalised Difference Vegetation Index (NDVI), is developed from satellite imagery of higher spatial and spectral resolution and corrects for some distortions in the reflected light caused by the particles in the air as well as the ground cover below the vegetation [140].

Urbanization: The availability of optimum environments for the development of the malaria transmitting anopheline populations become limited in urban areas resulting in reduced vector density, biting rates and transmission intensity[141-143]. Overall malaria infection rates are therefore lower in urban compared to rural areas of Africa[144,145].

Countries have different definitions of urbanisation that are rarely comparable across regions making their use in predictive modelling limited[145,71]. To develop a consistently defined surface of urbanisation, information from the Global Rural Urban Mapping Project (GRUMP)[75] and the Afripop project[72,71] was used. GRUMP urban extent grids distinguish urban and rural areas based on a combination of NOAA's Night-time lights dataset[147], settlements data and population counts. Population counts used were derived from GRUMP spatial population database based on areal weighted census input data [146] while settlements data sources include ESRI's Digital Chart of the World's Populated Places (DCW), Tactical Pilotage Charts (TPC) from Australian Defense Imagery and Geospatial Organization and some LandSAT-derived polygons [146,75]. To define urban extents, a border was defined around each set of contiguous lighted pixels whose total population count was greater than 5,000 persons. Because not all urban settlements are 'well-light' to be detected by satellite sensors, a buffer was drawn around settlement points to estimate spatial extents of the settlements. The GRUMP urban extent was further refined to produce a 'peri-urban' classification constrained by population density using the Afripop data[72]. Urban areas were defined as locations with a density of more than 1000 persons per km^2 with the rest of the GRUMP urban extent defined as peri-urban (Figure W3.1e).

Figure W3.1 Raster grid surfaces at 5 × 5 km of: a) Annual mean temperature; b) Temperature suitability index (TSI) for malaria transmission; c) Annual mean precipitation; d) Annual mean enhanced vegetation index (EVI); e) Urbanisation in 2010. The urbanisation surface derived from the Africa population surface was used as a template grid on which all other environmental grid were spatially matched. The light grey areas are those that were malaria free and the dark grey those that supported unstable transmission in 2000.

a)

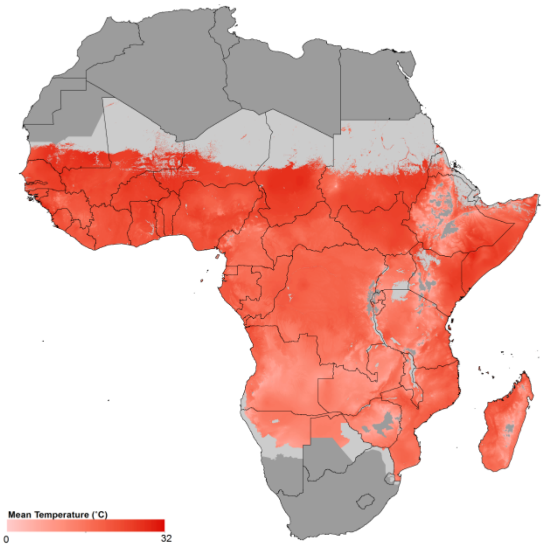

b)

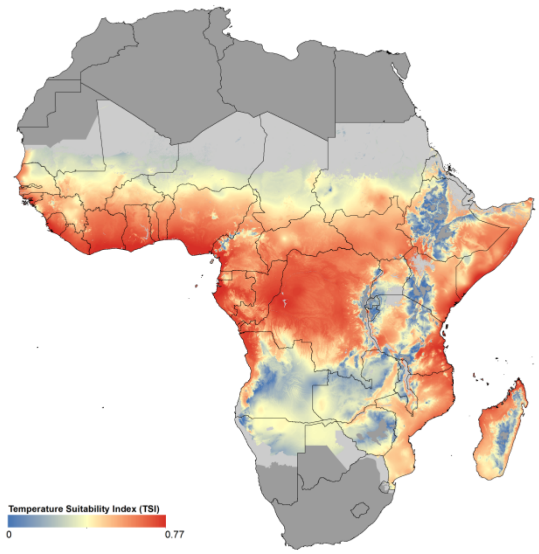

c)

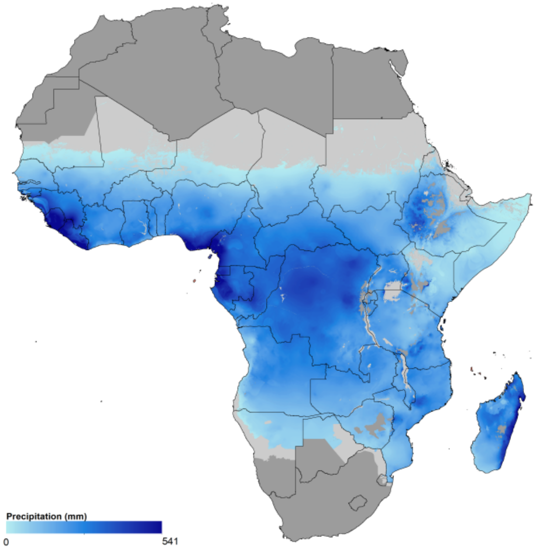

d)

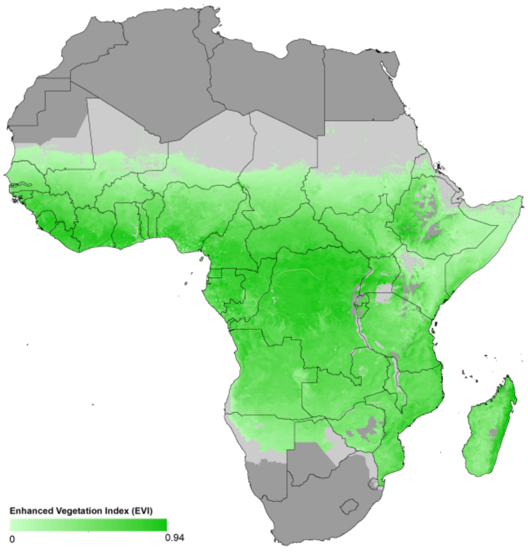

e)

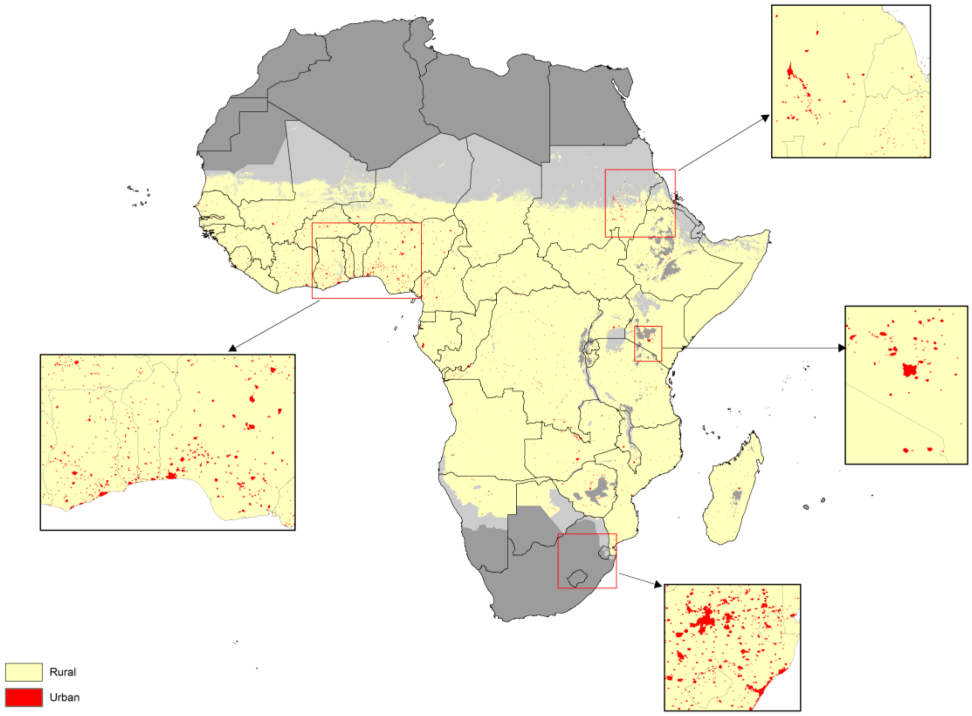

W3.3 Statistical selection of covariates

There are several approaches to selecting the best-fitting covariates in a statistical model [123,125,126, 148-150]. For this study, we used a strict covariate selection approach in which a total-sets analysis based on a generalized linear regression model was implemented in *bestglm* package in R[151-152]. Under this approach the best combination of covariates, which were those with the lowest value of the BIC statistic when regressed against *Pf*PR_{2-10}, were selected. The BIC penalty increases if the number of predictors is >7 so that compared to other information criterion, it selects the most parsimonious model[153].

To begin the selection process the values of the assembled covariates were extracted to each country-specific *Pf*PR_{2-10}survey location using ArcGIS 10 *Spatial Analyst* (ESRI Inc. NY, USA) tool. The data from the Islands were excluded in this analysis because several of the covariate surfaces did not have information for these islands (Cape Verde, São Tomé and Príncipe, Comoros and Mayotte) due to their small geographic size. For these islands, the data only was used to predict *Pf*PR_{2-10}. For each country, the total-set analysis was implemented on the full data from the period 1980-2012. The coefficients and 95% confidence intervals of the best-fit covariates from the total-set analysis are shown in Table W3.1. None of the selected covariates were selected in the best-fit model for Guinea Bissau, Eritrea and South Africa, Swaziland.

Table W3.1 The results of the total-set analysis by country showing the regression coefficients and the P-values of the best-fit covariates. The associations of precipitation, temperature suitability index (TSI), enhanced vegetation index (EVI) and urbanisation with *Pf*PR$_{2\text{-}10}$ were tested. Blank coefficients and P-values indicate that the covariate was not selected as part of the best-fit.

	Coefficient, P value			
**COUNTRY**	**TSI**	**Evi**	**Precipitation**	**Urban**
Angola		(0.799), (<0.001)		
Benin	(-1.879), (<0.001)	(-0.681), (0.015)		
Bioko & Annobon				
Botswana	(0.794), (<0.001)			(-0.059), (<0.001)
Burkina Faso	(1.612), (<0.001)	(1.902), (<0.001)	(-0.011), (<0.001)	(-0.099), (<0.001)
Burundi			(-0.005), (0.008)	
Cameroon	(0.297), (<0.001)	(0.665), (<0.001)		(-0.091), (0.0011)
Cape Verde				
Central African Republic	(-1.019), (0.300)	(-4.330), (0.009)	(0.0173), (0.037)	(-0.466), (0.002)
Chad	(2.190), (0.001)	(1.321), (0.001)	(0.003), (0.007)	(-0.076), (0.006)
Comoros				
Congo	(-3.277), (0.017)	(0.894), (0.035)	(-0.008), (0.006)	
Côte d'Ivoire			(0.004) , (<0.001)	(-0.222), (<0.001)
Djibouti				(-0.036), (<0.001)
DRC + Angola (Cabinda)	(0.300), (<0.001)		(0.002), (0.004)	(-0.269), (<0.001)
Equatorial Guinea			(0.002643590), (0.0182)	
Eritrea	0.0756 (0.0004)		0.0006 (0.016)	
Ethiopia	(0.059), (<0.001)			
Gabon			(0.00157738), (<0.001)	
Gambia	(-0.310), (<0.001)	(0.845), (<0.001)		
Ghana	(-0.209), (<0.001)			(-1.772), (<0.001)
Guinea	(-1.395), (<0.001)		(0.005), (<0.001)	(-0.106), (0.0013)
Guinea Bissau				
Kenya	(0.331), (<0.001)	(-0.352), (<0.001)	(0.003), (<0.001)	(-0.095), (<0.001)
Liberia	(-1.116), (<0.001)			(-0.096), (<0.001)
Madagascar	(0.164), (<0.001)		(0.001), (<0.001)	
Malawi				(-0.18), (<0.001)
Mali	(2.043), (<0.001)			(-0.227), (<0.001)
Mauritania		(0.336), (0.009)	(0.008), (0.668)	
Mayotte				
Mozambique	(0.582), (<0.001)		(0.002), (<0.001)	(-0.082), (<0.001)
Namibia		(0.500), (0.003)	(0.002), (0.003)	
Niger	(2.681), (<0.001)			(-0.236), (<0.001)
Nigeria				(-0.079), (0.001)
Rwanda			(-0.0006), (<0.001)	
São Tomé and Príncipe				
Senegal	(-0.310), (<0.001)	(0.845), (<0.001)		
Sierra Leone	(-2.510), (0.022)			
Somalia	(-0.116), (<0.001)		(0.002), (<0.001)	
South Africa				
South Sudan		(1.123), (<0.009))	(0.005), (<0.001))	
Sudan	(0.116), (<0.001)		(0.001), (<0.001)	
Swaziland				
Tanzania	(0.547), (<0.001)	(0.858), (<0.001)	(-0.001), (<0.001)	(-0.147), (<0.001)
Togo				(-0.140), (<0.001)
Uganda	(0.588),(<0.001)	(-0.325), (0.001432)	(0.002), (<0.001)	(-0.224), (<0.001)
Zambia	(0.993), (<0.001)	(0.531), (0.008)	(0.005), (<0.001)	(-0.088), (<0.001)
Zanzibar				
Zimbabwe	(0.407), (<0.001)		(0.002), (<0.001)	

W3.4 Model-based geostatistics (MBG)

The application of MBG methods in disease mapping is an established norm[154]. Where spatial and temporal data are available, MBG methods fit the data, in a way that the spatial (and temporal) covariance is used to generate samples of the predicted posterior distribution from which point estimates and the uncertainty around these estimates are computed simultaneously[154-156]. Normally, Bayesian inference is done using Markov Chain Monte Carlo (MCMC) algorithms[157]. MCMC approaches, although used widely, suffer from problems of convergence and dense covariance matrices which increase the computational time and cost significantly, especially where there are large data points spatially and temporally[158]. Recently, Integrated Nested Laplace Approximations (INLA) has been identified as an alternative algorithm for Bayesian inference[158]. The advantage of INLA-based approaches is mainly computational speed and can be undertaken in open source, easily adaptable R packages. Spatial and temporal analysis in INLA can be undertaken through the Stochastic Partial Differential Equations

(SPDE) approach[159] and the covariance functions are represented as Gaussian Markov Random Field (GMRF)[158,159].

W3.5 Model specification

A Bayesian hierarchical spatial-temporal model was implemented through SPDE approach using R-INLA library [R-INLA] to produce continuous maps of *PfPR*$_{2\text{-}10}$ at 1 × 1 km spatial resolution using data ranging from 1980-2012. The continuous indexed GF with covariance function was represented as a discretely indexed random process, that is, as a Gaussian Markov Random Field (GMRF)[159-161]. This is where an explicit link between Gaussian Field (GF) and GMRF formulated as a basis function is provided through Stochastic Partial Differential Equations (SPDE) approach[161-164]. The solution for SPDE can be expressed as

$$(k^2-\Delta)^{\alpha/2}(\tau x(u) = W(u), \quad u \in \circ^{\,d}, \quad \alpha = v + d/2, \quad \sigma^2 = \Gamma(\mathrm{v})/(\Gamma(\alpha)(4\pi)^{d/2}k^{2v}\tau^2)^{-1}$$
$$k>0, \ v>0, \qquad \text{(EquationW3.1)}$$

where W, is the spatial Gaussian white noise, Δ is the Laplacian, α controls the smoothness of the realization and τ controls the variance. The link between Matérn smoothness v and variance σ^2 is $\alpha = v + d/2$ and $\sigma^2 - \Gamma(\mathrm{v})(\Gamma(\alpha)(4\pi)^{d/2}k^{2v}\tau^2)^{-1}$, where d is the spatial dimension [165]. An approximation of this SPDE can be solved using a finite element method (FEM), which is a numerical technique for solving partial differential equations[43]. In this case, the spatio-temporal covariance function and dense covariance matrix of the GF are replaced by a neighbourhood structure and a sparse precision matrix respectively and together define a GMRF. A GMRF can be described as a spatial process that models spatial dependence of data observed at a spatial unit like grid or geographical region and it can be expressed as $u = (u_1 \mathrm{K}\ \mathrm{K}\ , u_n)'$ with $u \sim (\mu, Q^{-1})$. This is an n-dimensional GMRF with mean μ and a symmetrical positive definite precision matrix Q computed as the inverse of the covariance matrix [159]. Thus the density of u is given by

$$\pi(\mathrm{u}) = (2\pi)^{-n/2}|Q|^{1/2}\exp(-\frac{1}{2}(\mathrm{u}-\mu)'\,\mathrm{Q}(\mathrm{u}-\mu)) \qquad \text{(EquationW3.2)}$$

The sparse precision matrix Q offers computational advantage when making inference with GMRF. This is because the linear algebra operations can be performed using numerical methods for the sparse matrices which results in a considerable computational gain and this is further enhanced by using INLA algorithm for Bayesian inference[159-160]. The infinite-dimensional GRF is replaced with a finite-dimensional basis function representation

$$x(u) = \sum_{i=1}^{n}\psi_i(u)w_i \ , \qquad \text{(EquationW3.3)}$$

where w_i represents the weights and Ψ_i are piece-wise linear basis functions defined on a triangulation of the domain with n nodes which are defined as mesh in the code[161]. The basis functions are deterministic and are defined by each node in the triangulation while the stochastic property of the process is determined by the weights. The model used in this paper assumed non-stationary GRFs because environmental phenomena which are known to influence *PfPR*$_{2\text{-}10}$ are non-stationary in nature and therefore the distribution of *PfPR*$_{2\text{-}10}$ is non-stationary[166]. This non-stationary model was made possible by the flexible nature of SPDE models which allows modification of the SPDE rather than the covariance function to obtain the GFRs with other dependence structures other than the stationary Matérn covariance. The stationary isotropic Matérn covariance function, between locations u and v in $\circ^{\,d}$ is expressed as

$$C(u,v) = \frac{\sigma^2}{2^{v-1}\Gamma(v)}(k\|v-u\|)^v K_v(k\|v-u\|)\ , \qquad \text{(EquationW3.4)}$$

Where K_V is the modified Bessel function of the second kind, $\| \cdot \|$ denotes the Euclidean distance and order $v > 0$. $k > 0$ is a scaling parameter and σ^2 is the marginal variance. For the stationary model, k and v are constant in space. The parameter k is linked to the range p by the empirically derived relationship $p = \sqrt{8}/k$. k, here can be described as the range parameter presiding over the spatial dependence structure of the GRF [161]. For the non-stationary, τ and k space-dependent covariance parameters are introduced as functions of the spatial location $u, u \in D$, where D is the spatial domain. Therefore the modified SPDE becomes

$$(k(u)^2 - D)(\tau(u)x(u)) = W(u) \; , \; u \in ^\circ {}^2 , \qquad \text{(EquationW3.5)}$$

where x is a non-stationary GRF because τ and k vary by location and as the consequence the variance and correlation range vary by location. The non-stationary described above is defined on the mesh because it controls the local distance metric in the manifold. $\log \tau(u)$ and $\log k(u)$ can be defined as linear combination of basis function, where the basis functions $\left\{ \mathrm{B}_i^{(\cdot)}(\cdot) \right\}$ are smooth over the domain of interest.

$$\log(k(u)) = b_0^k(\mathrm{u}) + \sum_{k=1}^{p} b_k^k B_k(\mathrm{u}) \text{ and } \log(\tau(\mathrm{u})) = b_0^\tau(\mathrm{u}) + \sum_{k=1}^{p} b_k^\tau B_k(\mathrm{u}), \qquad \text{(EquationW3.6)}$$

For space-time models, the basis function can be represented as $x(u) = \sum_{i=1}^{n} \psi_i(u,t) w_i$ where each basis function is computed as a product of a spatial and a temporal basis function, $\psi_i(\mathrm{u,t}) = \psi_i^u(\mathrm{u})\psi_j^t(\mathrm{t})$, thus the space-time SPDE[165],

$$\frac{\vartheta}{\vartheta t}(\mathrm{k(u)}^2 - \Delta)^{\alpha/2}(\tau(\mathrm{u})\,\mathrm{x(u,t)}) = \mathrm{W(u,t)} \; , \; \mathrm{(u,t)} \in ^\circ \times \Omega \qquad \text{(EquationW3.7)}$$

Using this SPDE approach, the overall hierarchical space-time binomial model of the prevalence to malaria parasite were used denoted by

$$y(s,t) = z(s,t)\beta + \xi(s,t) + \varepsilon(s,t), \qquad \text{(EquationW3.8)}$$

This model is characterised by a GF *y(**s**, t)* built from covariate information *z(**s**, t)*, measurement error *ε(**s**, t)*, and a second order autoregressive dynamic model for the latent process *ξ(**s**, t)* with spatially correlated innovations *ω(**s**, t)*. The *Pf*PR$_{2\text{-}10}$ survey data were modelled as realizations of this spatial process (random field) changing in time. These realizations were used to make inference about the process and predict it at desired locations and at a specified time. This is where $y(s_i, t_j)$ was the realization of a spatial-temporal process representing the *Pf*PR$_{2\text{-}10}$ at the community location s_i, where $i = 1...n$, and year t_j where $j = 1...m$, $z(s_i,t_j) = (z_1(s_i,t_j) \mathrm{K} \; z_p(s_i,t_j))$ denotes the vector of p covariates for cluster s_i at time t_j, $\beta = (\beta_1 \mathrm{K} \;, \beta_p)'$ is the coefficient vector, $\varepsilon(s_i,t_j) : \; N(0,\sigma_\varepsilon^2)$ is the measurement error defined by the Gaussian white noise process, and $y(s_i,t_j)$ is the predicted posterior mean prevalence of the plasmodium parasite in cluster i at time j. In the model formulation the large scale component that depends on the covariates is defined as $Z(s_i,t_j)\beta$ while the measurement error variance or the nugget effect is σ_e^2. The realization of state process or the unobserved level of *Pf*PR$_{2\text{-}10}$ in this case is defined by $\xi(s_i,t_j)$ as a spatial-temporal GRF that changes in time as a second-order autoregressive function. Year instead of month was used

as the metric for time due to uncertainty around actual survey is several of the historical data and the added substantial complexity for the model.
The prior for the SPDE model by default are Gaussian. These priors are chosen heuristically to match the spatial scale of the MESH domain. The user can override the defaults by supplying a "hyper" parameter [165]. This is normally suitable when the dataset lacks enough information for the likelihood to fully identify the parameters for the prior distribution. In this paper the SPDE default priors were sufficient for the model.

W3.5.1 Constructing a suitable MESH
A finite element representation is used to outline the GRF as a linear combination of basis functions defined on a triangulation of the domain, say D. This is achieved by subdividing D into non-intersecting triangles meeting in at most common edge or corner, thus a *mesh*. The GRF in the triangulation is given by Equation (SI 3.3), where n is the total number of vertices, $\{\psi,(\mathrm{u})\}$ are the basis functions and $\{\omega_l\}$ are normally distributed weights [161,159].

The mesh function (*inla.mesh.create*) in INLA is used to create a Constrained Refined Delaunay Triangulation (CRDT). The overall effect of the triangulation construction is that, if desired, one can have smaller triangles, and hence higher accuracy of the field representation. However, this will have an effect on the computation of the model. There is therefore a need to balance the number of triangles and the computation time required. If the data points (cluster coordinates) are used to construct the mesh, a cut-off value is specified in the function represents which is the maximum distance in which data points are represented by a single vertex. If the boundary of the area domain is used to construct the mesh, (i.e using the function points.domain=border), then the mesh is constructed to cover the border of the domain using restrictions provided in other arguments. But if both data points and area domain (boundary) are used the restrictions are combined. In this model, the mesh was constructed using the boundary of the area domain. This method produces a mesh with regular size of triangles. A cut-off value was specified to avoid building many small triangles around PfPR$_{2\text{-}10}$ input locations. A reasonable offset value was used to specify the size of the inner and outer extensions around the data locations. The maximum edge value was used to specify the maximum allowed triangle edge lengths in the inner domain and in the outer extension. The inner maximum edge value was made small enough to allow the triangulation to support representing functions with small enough features, and typically smaller than the spatial correlation range of the model. Therefore this value was adjusted to fit the range of the area domain in the model[165].

A matrix was then constructed to link the $PfPR_{2\text{-}10}$ input locations to the triangles on the mesh defined by ŋ* as $= A(x+1)$ and in the inla code in the following *inla.spde.make.A* function. This makes each row in the matrix to have three non-zero elements since every data point is inside a triangle and the corresponding columns are expected to have non-zero elements. In order to obtain a square matrix for the model, the response was linked to the index of the random field, where the length of the index vector was the same as the length of the projection matrix. In order to estimate the intercept, the stack function introduces a vector of ones in the matrix and this is later removed in the formula[165].

W3.5.2 Model outputs
The continuous PfPR$_{2\text{-}10}$ predictions are shown in Figure W3.2. Table W3.2 shows country estimation of population at risk by PfPR$_{2\text{-}10}$endemicity class in 2000 and 2010. Countries are sorted by the baseline (2000) PAPfPR$_{2\text{-}10}$.

For CAR, Congo, Mauritania available data were not sufficient to make reliable predictions in any of the two prediction years. For Burundi, Niger and Mayotte data were not sufficient to make predictions for 2010. These countries were excluded in the analysis of change between 2000 and 2010. In DRC and Chad data were not sufficient to make predictions in 2000 but we assumed that no major change in malaria risk would have occurred between the two time points due to the very low coverage of malaria interventions by 2010[168] and have used the 2010 map to also represent transmission in 2000.

Figure W3.2 The predicted 1 × 1 spatial resolution maps of Africa of: a) continuous *Pf*PR$_{2\text{-}10}$ in 2000; b) continuous *Pf*PR$_{2\text{-}10}$ in 2010. The dark and light grey areas are the limits of malaria free and unstable transmission respectively for each year

a) **b)**

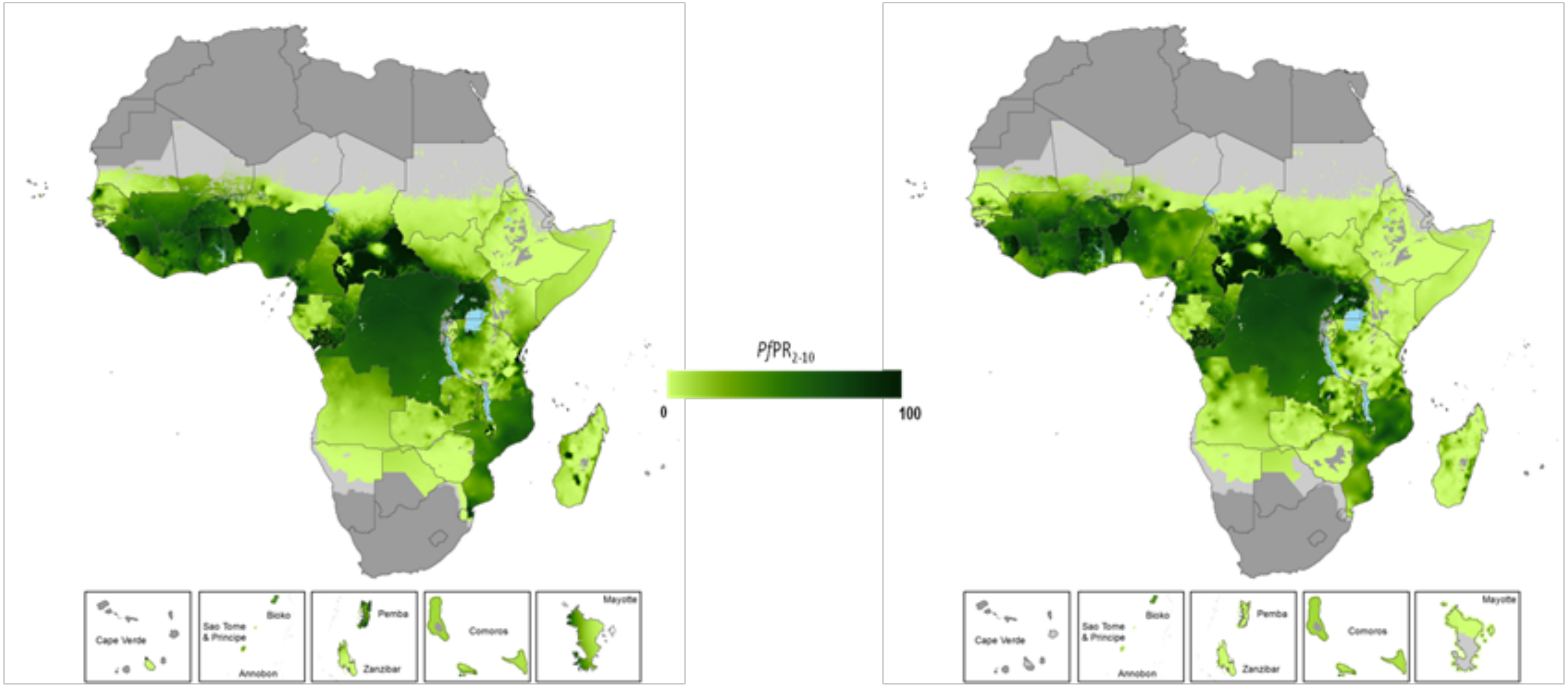

Table W3.2 The population at risk (in millions) by endemicity class for all MECs in Africa and by country and territory for the years 2000 and 2010. Countries are sorted according to increasing PA*Pf*PR$_{2-10}$ in 2000.

		Malaria free		Unstable		<1% *Pf*PR$_{2-10}$		1% to <5% *Pf*PR$_{2-10}$		5% to <10% *Pf*PR$_{2-10}$		>10% to 50% *Pf*PR$_{2-10}$		>50% to 75% *Pf*PR$_{2-10}$		>75% to 100% *Pf*PR$_{2-10}$	
**REGION**	**COUNTRY**	**2000**	**2010**	**2000**	**2010**	**2000**	**2010**	**2000**	**2010**	**2000**	**2010**	**2000**	**2010**	**2000**	**2010**	**2000**	**2010**
**Central Africa**		**3.27**	**4.31**	**0.48**	**0.71**	**1.11**	**2.37**	**2.71**	**9.25**	**3.74**	**5.44**	**35.28**	**40.87**	**36.52**	**47.25**	**5.78**	**11.54**
	Angola	0.01	0.01	0.23	0.37	0	0.85	1.28	7.22	2.73	3.3	9.47	6.81	0	0.24	0	0
	Bioko & Annobon	0.02	0.03	0	0	0	0	0	0	0	0	0.07	0.13	0.03	0.01	0	0
	Cameroon	0.22	0.25	0	0	0	0	0	0.01	0.01	0.63	11.95	15.87	2.94	2.39	0.41	0.27
	CAR																
	Chad	0	0	0.25	0.34	1.11	1.51	1.41	1.93	0.67	0.92	1.51	2.06	1.30	1.77	1.96	2.68
	Congo																
	DRC	3.01	4.01	0	0	0	0	0	0	0	0	11.33	15.09	32.03	42.64	3.18	4.24
	Equatorial Guinea	0	0	0	0	0	0	0	0	0	0	0.01	0.01	0.13	0.2	0.23	0.3
	Gabon	0	0	0	0	0	0	0.02	0.07	0.32	0.47	0.88	0.89	0.03	0	0	4.05
	São Tomé and Príncipe	0.01	0.01	0	0	0	0.01	0	0.02	0.01	0.12	0.06	0.01	0.06	0	0	0
**East Africa**		**26.14**	**33.47**	**10.47**	**13.13**	**47.99**	**81.33**	**39.46**	**62.46**	**19.26**	**17.58**	**39.38**	**35.49**	**7.77**	**11.02**	**18.17**	**15.51**
	Burundi																
	Comoros	0	0	0	0	0	0	0.01	0.01	0.01	0.02	0.02	0.01	0	0	0	0
	Djibouti	0	0	0.64	0.79	0.06	0.08	0	0	0	0	0	0	0	0	0	0
	Eritrea	0.01	0.02	0.88	1.26	1.59	3.46	0.81	0.49	0.16	0	0.19	0	0.01	0	0	0
	Ethiopia	17.35	22.13	0.67	0.84	36.45	45.45	8.63	11.62	1.54	2.22	0.81	0.55	0	0	0	0
	Kenya	3.7	4.71	0.26	0.33	3.49	10.05	3.5	10.16	3.51	3.56	16.05	10.96	0.59	0.55	0	0
	Mayotte	0	0.04	0	0.1	0.12	0.06	0.03	0.01	0	0	0	0	0	0	0	0
	Rwanda	2.89	3.72	0	0	2.3	4.22	2.87	2.54	0	0.08	0	0.03	0	0	0	0
	Somalia	0.03	0.03	0.42	0.52	0	0.09	0.38	6.89	5.81	1.77	0.75	0	0	0	0	0
	South Sudan	0	0.01	0	0	0.12	0.18	1.42	3.33	2.09	1.74	2	3.04	0.82	1.16	0.09	0.26
	Sudan	0	0	7.59	9.28	2.7	7.34	14.57	14.92	2.41	1.79	0.17	0.13	0	0	0	0
	Tanzania	1.24	1.57	0.01	0.01	1.11	9.47	4.22	8.85	3.65	5.79	18.94	16.71	2.55	0.28	1.26	0.12
	Uganda	0.92	1.24	0	0	0.01	0.46	2.64	3.32	0.03	0.47	0.1	3.74	3.68	9.03	16.82	15.13
	Zanzibar	0	0	0	0	0.04	0.47	0.38	0.32	0.05	0.14	0.35	0.32	0.12	0	0	0
**Southern Africa**		**46.08**	**54.91**	**1.66**	**4.6**	**15.4**	**19.63**	**10.46**	**11.24**	**5.27**	**5.95**	**25.38**	**31.26**	**10.73**	**11.67**	**1.66**	**0.56**
	Botswana	0.9	1.03	0	0.72	0.13	0	0.62	0.14	0.1	0.11	0	0	0	0	0	0
	Madagascar	1.01	1.32	0.01	0.01	4.74	8.95	4.12	4.84	1.84	2.32	3.41	3.02	0.08	0.13	0.07	0
	Malawi	0.01	0.01	0	0	0.09	0	0.17	0.02	0.42	0.01	8.21	10.59	1.63	4.09	0.68	0.14
	Mozambique	0.01	0.01	0.05	0.07	0	0.65	0	0.8	0	0.94	8.16	13.5	8.98	7.05	0.91	0.26
	Namibia	0.22	0.28	0.34	0.46	0.26	0.47	0.96	1	0.09	0.06	0	0	0	0	0	0
	South Africa	41.67	47.01	1.25	2.98	1.57	0.04	0.11	0	0.04	0	0.03	0	0.01	0	0	0
	Swaziland	0.19	0.81	0	0.34	0.87	0.02	0	0	0	0	0	0	0	0	0	0
	Zambia	0	0	0.01	0.02	0.06	3.36	2.81	3.13	2.3	2.21	4.98	3.79	0.03	0.4	0	0.16
	Zimbabwe	2.07	4.44	0	0	7.68	6.14	1.67	1.31	0.48	0.3	0.59	0.36	0	0	0	0
**West Africa**		**0**	**5.97**	**0.68**	**1.07**	**0.39**	**5.41**	**0.9**	**7.8**	**2.97**	**5.47**	**78.58**	**172.54**	**124.37**	**76.08**	**13.55**	**9.85**
	Benin	0	0	0	0	0	0.1	0.16	0.45	0.57	0.99	2.33	3.27	0.57	0.76	2.89	3.25
	Burkina Faso	0	0	0.06	0.09	0	0	0	0	0	0	2.77	6.51	8.39	9.70	1.06	0.06
	Cape Verde	0	0.16	0	0.11	0.22	0	0	0	0	0	0.01	0	0.01	0	0	0
	Côte d'Ivoire	0	0	0	0	0	0	0	0	0	0	3.36	4.59	11.59	13.45	1.62	1.68
	Gambia	0	0	0	0	0	0.06	0	0.8	0.14	0.51	1.13	0.31	0	0.01	0	0
	Ghana	0	0	0	0	0	0	0	1.15	0.01	2.12	10.52	9.69	6.6	10.13	1.93	1.16
	Guinea	0	0	0	0	0	0	0	0	0	0	3.32	4.29	3.77	5.63	1.23	0
	Guinea Bissau	0	0	0	0	0.17	0.44	0.46	0.81	0.12	0.07	0.46	0.17	0.01	0.01	0	0
	Liberia	0	0	0	0	0	0	0	0	0	0	2.66	3.7	0.16	0.22	0	0
	Mali	0	0	0.43	0.57	0	0	0.06	0.14	0.05	0.35	5.54	7.94	5.2	5.56	0	0.79
	Mauritania																
	Niger																
	Nigeria	0	0	0.14	0.22	0	0	0	0	0	0.07	38.78	130.63	82.11	27.31	2.52	0
	Senegal	0	0	0.05	0.08	0	4.81	0.22	4.45	2.08	1.36	6.41	1.42	0.57	0.16	0.1	0.05
	Sierra Leone	0	5.81	0	0	0	0	0	0	0	0	1.29	0	0.7	-2.82	2.12	2.82
	Togo	0	0	0	0	0	0	0	0	0	0	0	0	4.69	5.96	0.08	0.04
**All MECS in Africa**		**75.49**	**98.66**	**13.29**	**19.51**	**64.89**	**108.74**	**53.53**	**90.75**	**31.24**	**34.44**	**178.62**	**280.14**	**179.39**	**146.02**	**39.16**	**37.46**

W3.5.3 Model Validation

As a first step to understanding the uncertainty around the predictions of *Pf*PR_{2-10} using the Bayesian geostatistical model, the continuous mean map and the endemicity class map for each year were accompanied by estimates of the posterior standard deviation (Figure W3.3).The percentage of population by standard deviation to posterior mean *Pf*PR_{2-10} is shown in Table W3.3. In addition, a spatially and temporally representative validation set of *Pf*PR_{2-10} survey data were selected using a sampling algorithm [167] which declusters over space and time. This algorithm defined the Thiessen polygons around each survey location with each data point having a probability of selection proportional to the area of its Thiessen polygon and taking time factor into place. This allowed for data located in densely surveyed regions to have a lower probability of selection than those in sparsely surveyed regions setting a high threshold for model performance. The annual predictions were then repeated in full using the remaining data points to predict *Pf*PR_{2-10} at the validation locations. The ability of the model to predict point-values of *Pf*PR_{2-10} at unsampled locations was quantified using four simple summary statistics: the correlation coefficient between the predicted and actual set; the mean prediction error (MPE); the mean absolute prediction error (MAE); and the root mean square error (RMSE). The correlation coefficient provides a simple measure of linear association between the data and prediction sets, the ME provides a measure of the model bias, and the MAE and RMSE is a measure of the average accuracy of individual predictions. The results of the model validation by country are provided in Table W3.4.

Figure W3.3 Maps of number of the standard deviations from the predicted mean *Pf*PR_{2-10} in: a) 2000; and b) 2010. The dark and light grey areas are the limits of malaria free and unstable transmission respectively for each year.

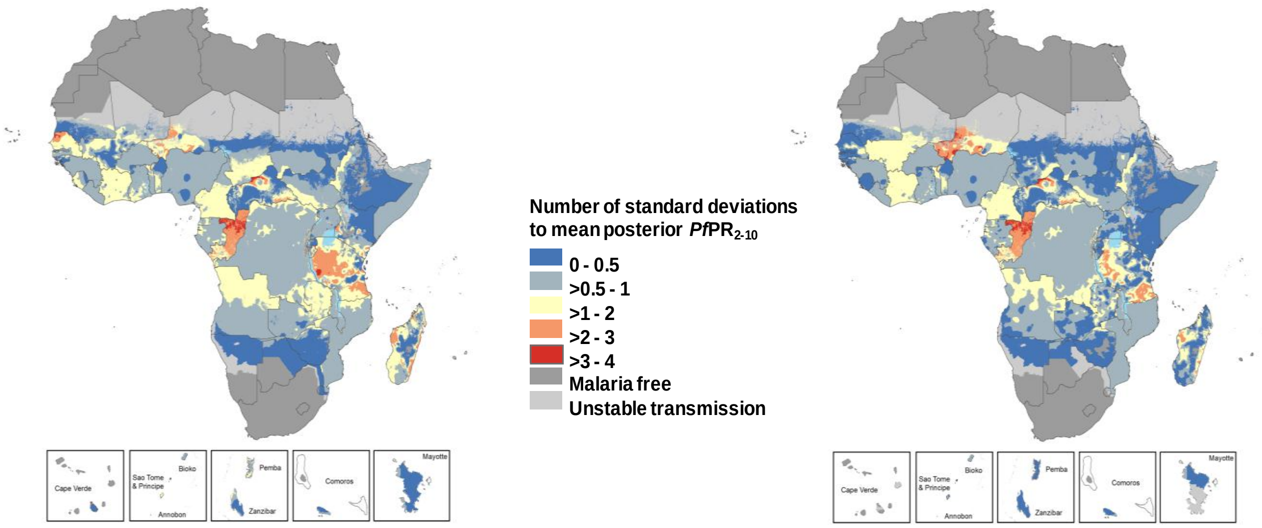

Table W3.3 The percentage of population at risk by the standard deviation from the predicted mean *Pf*PR_{2-10} in 2000 and 2010.

	Proportion of population by standard deviation (SD) to the mean *Pf*PR_{2-10}							
	2000				2010			
COUNTRY	0 -1 SD	>1 - 2 SD	>2 - 3 SD	>3 - 4 SD	0 -1 SD	>1 - 2 SD	>2 - 3 SD	>3 - 4 SD
Angola	58.2	41.8	0.0	0.0	77.9	22.1	0.0	0.0
Benin	62.9	35.4	1.5	0.3	69.7	28.8	1.3	0.2
Bioko & Annobon	100.0	0.0	0.0	0.0	100.0	0.0	0.0	0.0
Botswana	100.0	0.0	0.0	0.0	100.0	0.0	0.0	0.0
Burkina Faso	96.0	4.0	0.0	0.0	99.9	0.1	0.0	0.0
*Burundi	23.8	44.3	16.6	15.2				
Cameroon	36.8	63.2	0.0	0.0	50.4	49.6	0.0	0.0
Cape Verde	94.6	2.4	1.5	1.5				
*Central African Republic (CAR)								
*Chad	96.1	3.9	0.0	0.0	96.1	3.9	0.0	0.0
Comoros	99.6	0.4	0.0	0.0	99.6	0.4	0.0	0.0
*Congo								
Côte d'Ivoire	9.3	90.7	0.0	0.0	6.8	93.2	0.0	0.0
Djibouti	100.0	0.0	0.0	0.0	100.0	0.0	0.0	0.0
*Democratic Republic of Congo (DRC)	87.4	12.6	0.0	0.0	87.4	12.6	0.0	0.0
Equatorial Guinea	47.9	52.1	0.0	0.0	53.4	46.6	0.0	0.0
Eritrea	100.0	0.0	0.0	0.0	100.0	0.0	0.0	0.0
Ethiopia	96.7	3.2	0.1	0.0	97.7	2.3	0.0	0.0
Gabon	51.9	48.1	0.0	0.0	51.8	48.2	0.0	0.0
Gambia	99.9	0.1	0.0	0.0	100.0	0.0	0.0	0.0
Ghana	81.5	18.5	0.0	0.0	98.0	2.0	0.0	0.0
Guinea	99.1	0.9	0.0	0.0	99.2	0.8	0.0	0.0
Guinea Bissau	90.0	10.0	0.0	0.0	95.2	4.8	0.0	0.0
Kenya	65.3	19.4	15.4	0.0	99.4	0.6	0.0	0.0
Liberia	100.0	0.0	0.0	0.0	100.0	0.0	0.0	0.0
Madagascar	59.6	26.3	14.1	0.0	72.9	24.4	2.7	0.0
Malawi	94.0	6.0	0.0	0.0	94.1	5.9	0.0	0.0
Mali	81.4	18.6	0.0	0.0	6.9	93.1	0.0	0.0
*Mauritania								
Mayotte	99.8	0.2	0.0	0.0	100.0	0.0	0.0	0.0
Mozambique	95.3	4.7	0.0	0.0	99.5	0.5	0.0	0.0
Namibia	100.0	0.0	0.0	0.0	100.0	0.0	0.0	0.0
*Niger	26.1	56.2	17.8	0.0				
Nigeria	100.0	0.0	0.0	0.0	100.0	0.0	0.0	0.0
Rwanda	100.0	0.0	0.0	0.0	100.0	0.0	0.0	0.0
São Tomé and Príncipe	100.0	0.0	0.0	0.0	100.0	0.0	0.0	0.0
Senegal	15.9	62.5	20.2	1.4	97.8	2.2	0.0	0.0
Sierra Leone	44.7	55.3	0.0	0.0	100.0	0.0	0.0	0.0
Somalia	100.0	0.0	0.0	0.0	100.0	0.0	0.0	0.0
South Africa	100.0	0.0	0.0	0.0	100.0	0.0	0.0	0.0
South Sudan	84.3	15.7	0.0	0.0	95.0	5.0	0.0	0.0
Sudan	98.9	1.1	0.0	0.0	99.2	0.8	0.0	0.0
Swaziland	100.0	0.0	0.0	0.0	100.0	0.0	0.0	0.0
Tanzania	20.5	47.5	31.5	0.5	56.9	36.6	6.5	0.0
Togo	80.3	19.7	0.0	0.0	73.8	26.2	0.0	0.0
Uganda	100.0	0.0	0.0	0.0	100.0	0.0	0.0	0.0
Zambia	68.9	31.1	0.0	0.0	95.0	5.0	0.0	0.0
Zanzibar	76.0	24.0	0.0	0.0	52.1	47.9	0.0	0.0
Zimbabwe	94.9	5.1	0.0	0.0	95.9	4.1	0.0	0.0
**Total**	78.2	17.6	4.0	0.2	85.4	12.9	1.6	0.1

*Data was sufficient to predict *Pf*PR_{2-10} for 2000 only for Niger and Burundi; and 2010 for Chad and DRC. There was insufficient data to predict to either years in CAR, Congo and Mauritania.

Table W3.4 The *Pf*PR_{2-10} full space-time model validation statistics by country based on the actual observation and predictions to a 10% holdout dataset per country. R= the linear correlation coefficient; MPE = the mean prediction error; and the MAPE=mean absolute prediction error.

COUNTRY	RMSE	MPE	MAPE	R
Angola	5.43	-1.52	3.42	0.98
Benin	16.53	11.98	12.14	0.93
Bioko & Annobon	1.31	-0.08	1.09	0.99
Botswana	1.18	-0.12	0.81	0.99
Burkina Faso	12.91	0.80	8.57	0.84
Burundi	6.77	-2.46	4.71	0.62
Cameroon	13.10	0.76	9.18	0.80
Cape Verde	0.99	-0.79	0.88	0.67
*Central African Republic				
Chad	5.05	-0.46	3.49	0.89
Comoros	1.03	-0.80	0.87	0.81
*Congo				
Côte d'Ivoire	8.26	0.24	5.83	0.94
Djibouti	1.06	-0.35	0.56	0.70
DRC + Cabinda (Angola)	17.79	-0.61	11.26	0.84
Equatorial Guinea	1.33	0.55	1.05	0.99
Eritrea	2.87	0.74	1.34	0.91
Ethiopia	3.11	0.00	0.01	0.94
Gabon	15.12	3.53	6.59	0.86
Gambia	7.08	-0.06	4.47	0.82
Ghana	7.40	0.99	4.72	0.97
Guinea	15.95	-3.60	12.36	0.78
Guinea Bissau	7.52	2.37	3.44	0.91
Kenya	4.57	0.26	1.32	0.96
Liberia	7.41	-1.26	5.44	0.93
Madagascar	2.57	-0.04	1.35	0.89
Malawi	11.90	-1.49	8.80	0.84
Mali	7.70	1.04	4.78	0.98
*Mauritania				
Mayotte	2.74	1.05	1.57	0.86
Mozambique	7.90	-0.45	5.29	0.92
Namibia	7.34	-1.14	3.58	0.84
Niger	4.48	-2.05	2.10	0.67
Nigeria	14.76	-3.75	8.02	0.87
Rwanda	2.22	0.16	1.16	0.76
São Tomé and Príncipe	1.86	0.71	1.32	0.99
Senegal	7.08	-0.06	4.47	0.82
Sierra Leone	4.99	-0.90	3.26	0.97
Somalia	9.83	0.12	4.86	0.72
South Africa	0.88	0.18	0.40	0.78
South Sudan	5.09	1.27	3.51	0.98
Sudan	4.45	0.10	2.21	0.76
Swaziland	0.08	0.01	0.02	0.99
Tanzania	8.94	-0.18	5.39	0.94
Togo	16.43	-6.33	11.43	0.71
Uganda	11.78	-0.07	6.71	0.87
Zambia	10.71	-0.20	5.69	0.88
Zanzibar	4.19	1.16	2.36	0.99
Zimbabwe	4.49	0.13	1.78	0.90

*Data was sufficient to predict *Pf*PR_{2-10} for 2000 only for Niger and Burundi; and 2010 for Chad and DRC. There was insufficient data to predict to either years in CAR, Congo and Mauritania.

W3.6 Rationale for not selecting data from borders with neighbouring countries

We paid a particular attention to the issue of borrowing border data. Conceptually it makes sense to borrow data from borders on the assumption that transmission on one side of the border is likely to be a function of transmission on the other side. However, what is clear from the published literature[168,169] is the large disparity in the extent of malaria control and socio-economic progress and therefore the likely reductions in transmission, between neighbouring countries in Africa.

To reliably understand the effect of borrowing data from borders, the best test case is using border data to predict risk in a country where large reductions of malaria have been achieved and where evidence exists that transmission is extremely low, but borders a country of relatively high transmission and low levels of control. We used Mozambique (high transmission, low control) and Swaziland (very low transmission, high level of control) for this illustrative experiment. In Swaziland the MIS of 2010 showed that out 5567 people who were tested, only 1 was positive for malaria.

When we made predictions without using border data the risk map largely corresponded with the reported data. When additional data from a 100 km buffer into Mozambique was used, the northeastern part of Swaziland showed highly elevated levels of risk (see images below), which is contrary to any available evidence from the country (Kunene et al 2011). Therefore, making predictions for a country using only data from that country was a more parsimonious approach that will prevent over or under estimation of risk along the borders of a country.

Figure W 3.4 Continuous *Pf*PR_{2-10} maps of Swaziland for 2010 produce: a) with data borrowed from the border with Mozambique and analysis done as part of a Southern Africa regional tile; b) without any border data included and using only data from Swaziland analysed separately

a) b)

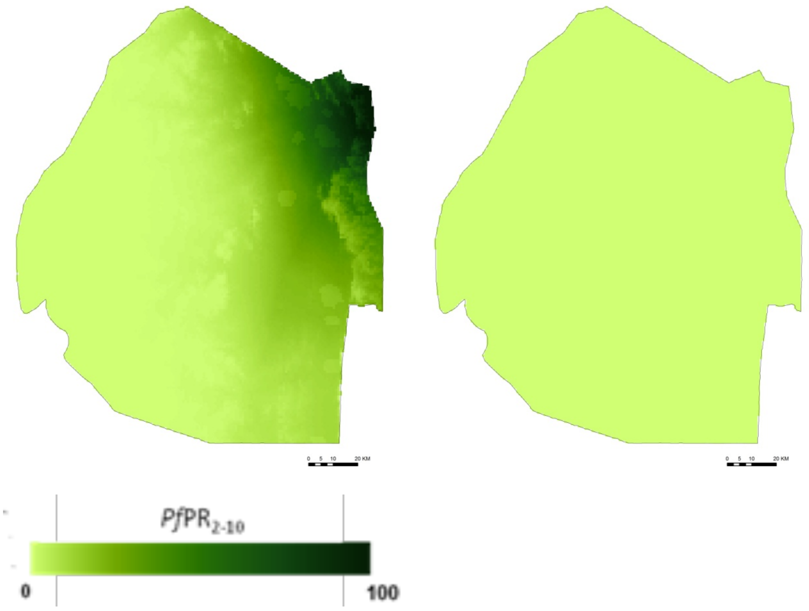

W4 References

1. Macdonald G. The analysis of equilibrium in malaria. *Trop Dis Bull* 1952; **49**: 813-828.

2. Pampana EJ. *A textbook of malaria eradication*. Second edition. London: Oxford University Press, 1969.

3. Pull JH. Malaria surveillance methods, their uses and limitations. *Am J Trop Med & Hyg* 1972*;* **21**: 651-655.

4. Ray AP, Beljaev AE. Epidemiological surveillance: a tool for assessment of malaria and its control. *J Comm Dis* 1984 ; **16**: 197-207.

5. Hay SI, Smith DL, Snow RW. Measuring malaria endemicity from intense to interrupted transmission. *Lancet Infect Dis* 2008; **8**: 369-378.

6. Cohen JM, Moonen B, Snow RW, Smith DL. How absolute is zero? An evaluation of the historical and current definitions of malaria elimination. *Malar J* 2010; **9**: 213.

7. Feachem RGA, Phillips AA, Hwang J, Cotter C, Weilgosz B, *et al*. Shrinking the malaria map: progress and prospects. *Lancet* 2010a; **376**: 1566-1578.

8. Snow RW, Amratia P, Kabaria CW, Noor AM, Marsh K. The changing limits and incidence of malaria in Africa: 1939-2009. *Adva Parasitol* 2012; **78**: 169-262.

9. Carter R, Mendis KN. Evolutionary and historical aspects of the burden of malaria.*Clin Microbiol Rev* 2002; **15**: 564-594.

10. Russell PF. World-wide malaria distribution prevalence and control. *Am J Trop Med & Hyg* 1956; **6**: 937-965.

11. World Health Organization-Spanish Morocco. *Information on the malaria control programme in Spanish Morocco*. World Health Organization, Lagos Conference, WHO/MAL/126-6-1,1955.

12. International Association for Medical Assistance to Travelers, IAMAT (2013).

13. Boubidi SC, Gassen I, Khechache Y, Lamali K, Tchicha B, *et al*. *Plasmodium falciparum* malaria, Southern Algeria, 2007. *Emerg Infect Dis* 2010*;* **16**: 301-303.

14. Hamon J & Dufour G. La lutte antipaludique a la Réunion. *Bull World Health Org* 1954; **11**: 525-556.

15. Picot H. Prévention contre la réintroduction du paludisme a la Réunion en 1972-1973. *B Soc Pathol Exot* 1976; **69**: 134-140.

16. Ross R. *Report on the Prevention of Malaria in Mauritius*. London: Waterlow and Sons Limited, 1908.
17. Balfour-Kirk J. Malaria in Mauritius. *Trop Dis Bull* 1934; **31**: 521-537.

18. Noor AM, Mohamed MB, Mugyenyi CK, Osman OA, Guessod HH, *et al*. Establishing the extent of malaria transmission and challenges facing pre-elimination in the republic of Djibouti.*BMC Infect Dis* 2011;**11**:121.

19. De Meira MTV. Relatório anual da missão permanente de estudo e combate de endemias de Cabo Verde (1961). *An Inst Hig Med Trop (Lisb)* 1963; **20**: 341-351.

20. Cambournac FJC, Vieira HSR, Coutinho MA, Soares FA, Soares AB, Janz GJ. Note sur l'éradication du paludisme dans l'ile de Santiago (République du Cap-Vert). *An Inst Hig Med Trop (Lisb)* 1984; **10**: 23-34.

21. Alves J. Programme National de Lutte contre le paludisme. Plan d'Action 1994-98. Ministère de la Santé, République du Cap-Vert, 1994.

22. Alves J, Roque AL, Cravo P, Valdez T, Jelinek T,*et al*. Epidemiological characterization of *Plasmodium falciparum* in the Republic of Cabo Verde: implications for potential large scale re-emergence of malaria. *Malar J* 2006; **5**: 32.

23. Ali Halidi ME. Paludisme à Mayotte: passé, présent, futur. *Cahier Santé* 1995; **5**: 362-367

24. Julvez J, Michault A, Galtier J, Ali Halidi E, Vidal JM. Evaluation seroepidemologique du paludisme a Mayotte. *B Soc Pathol Exot* 1990; **83**: 658-668.

25. Receveur MC, Roussin C, Vatan R, de Montéra AM, Sissoko D, *et al*. Bilan du paludisme à Mayotte. Épidémiologie, diagnostic, prévention et traitement. *B Soc Pathol Exot* 2004; **97**: 265-267.

26. Solet JL, Balleydier E, Quatresous I, Sanquer MA, Gabrie P, *et al*. Situation épidémiologique du paludisme à Mayotte, France en 2005 et 2006. *Bulletin Épidémiologique Hebdomadaire* 2007; **48**: 407- 409.

27. http://ars.ocean-indien.sante.fr/ResIST-Reseau-de-Surveillance.100039.0.html (accessed Jan, 2013).

28. Joaquim A, de Mesquila VHB. Contribuicao para o estudo do sezonismo em S Tomé. *An Inst Hig Med Trop (Lisb)* 1946; **22**: 13-66.

29. World Health Organization-Sao Tome and Principe.*Information on the malaria control programme in São Tomé and Príncipe*. World Health Organization, Lagos Conference, WHO/MAL/126-4-5, 1955.

30. Ceita JGV. O projecto de eradication do paludismo na Repûblica Democrática de São Tomé e Prínicpe. Repûblica Democrática de São Tomé and Príncipe Missão Eradication Paludismo, October 1981.

31. Lee PQ, Liu CT, Rampao HS, Rosario VE, Shaio ME. Pre-elimination of malaria on the island of Principe. *Malar J* 2010; **9**: 26.

32. Teklehaimanot HD, Teklehaimanot A, Kiszewski A, Rampao HS, Sachs JD. Malaria in São Tomé and Príncipe: on the brink of elimination after three years of effective antimalarial measures. *Am J Trop Med & Hyg* 2009; **80**: 133-140.

33. Tseng LF, Chang WC, Ferreira MC, Wu CH, Rampao HS, *et al*. Rapid control of malaria by means of indoor residual spraying of alphacypermethrin in the Democratic Republic of São Tomé and Principe. *Am J Trop Med & Hyg* 2008; **78**: 248-250.

34. Delfini FL. Malaria Eradication Programme, Zanzibar and Pemba. World Health Organization. AFR/MAL/104, 1969.

35. Schwartz E, Pener H, Issa MS, Golenser J. An overview of the malaria situation in Zanzibar . *J Comm Health* 1997; **22**: 33-44.

36. Zanzibar Malaria Control Program (2009). *Malaria elimination in Zanzibar: a feasibility assessment*. URL: http://www.malariaeliminationgroup.org (accessed Feb, 2012).

37. Raynal J. Enquête sanitaire à la Grande Comores 1925, observation de paludisme a forme épidémique. *B Soc Pathol Exot* 1928a ; **21**: 132-141.

38. World Health Organization-Comoros. *Informations sur le programme de lutte antipaludique dans l'archipel des Comores*. World Health Organization, Lagos Conference, WHO/MAL/126-2-4, 1955.

39. Blanchy S, Benthein F, Sabatinelli G. Epidémiologie du paludisme en République Fédérale Islamique des Comores. *Cahiers O.R.S.T.O.M. ser Entomol Méd Parasit* 1987; **25** numero special: 45-52.

40. Programme National de Lutte Contre le Paludisme – Comores (2009). *Plan stratégique de lutte contre le paludisme: 2007-2014*. Programme National de Lutte contre de Paludisme, Direction de la lutte contre les endémies et épidémies, Ministre de Sante, Union de Comores, March 2009.

41. Brink CJH.Malaria control in the northern Transvaal. *S Afr Med J* 1958; **32**: 800-808.

42. Ministry of Health .*Botswana Roll Back Malaria Strategic Plan 2002-2005*. Ministry of Health, Botswana, November 2001.

43. Sharp BL, Le Sueur D. Malaria in South Africa- the past, the present and selected implications for the future. *S Afr Med J* 1996; **86**: 83–89.

44. Craig MH, Kleinschmidt I, Le Seuer D, Sharp BL. Exploring 30 years of malaria case data in KwaZulu-Natal, South Africa: Part II. The impact of non-climatic factors. *Trop Med & Int Health* 2004; **9**: 1258-1266.

45. Hansford CF. Malaria control in the Northern Transvaal. *S Afr Med J* 1974; **48**: 1265-1269.

46. Kleinschmidt I, Sharp BL, Clarke, CPY, Curtis B, Fraser C. Use of Generalized Linear Mixed Models in the spatial analysis of small-area malaria incidence rates in KwaZulu Natal, South Africa.*Ame J Epi* 2001; **153**: 1213-1222.

47. De Meillon B. Malaria survey of South-West Africa. *Bull World Health Org* 1951; **4**: 333–417.

48. Ministry of Health and Social Services. *National Policy and Strategy for Malaria Control*. Ministry of Health and Social Services Namibia, November 1995.

49. Noor AM, Alegana VA,Kamwi R, Hansford CF, Ntomwa B, *et al*. Malaria control and the intensity of *Plasmodium falciparum* transmission in Namibia 1969-1992. *PLoS One* 2013; **8**: e63350.

50. Snow RW, Alegana VA, Makomva K, Reich A, Uusiku P, *et al*. *Estimating the distribution of malaria in Namibia in 2009: assembling the evidence and modelling risk.* Ministry of Health and Social Services, Republic of Namibia and Malaria Atlas Project, May 2010.

51. Ministry of Health and Social Services. *Namibia malaria indicator survey 2009*. National Vector Borne Diseases Control Programme, Ministry of Health and Social Services, Namibia,2009.

52. Bechuanaland Protectorate. Annual Medical and Sanitary Reports for the Protectorate for the year 1963. Gaborone: Government Printers,1963.

53. Franco de ALT, Roche SM, Ariaratnam V, Joia HS, Chinien V. Malaria situation in Botswana. Report on Evaluation Mission WHO Consultant Team, September-November 1984.

54. Ministry of Health. *Botswana Malaria Programme performance review report*. Department of Public Health, Ministry of Health, 2009; pages 1-60,2009.

55. Thomson JG. Section of epidemiology and state medicine: endemic and epidemic malaria in Southern Rhodesia. *Proc R Soc Med* 1929; **22**: 1051-1058.

56. Taylor P, Mutambu SL.A review of the malaria situation in Zimbabwe with special reference to the period 1972-1981.*Trans Royal Soc of Trop Med & Hyg* 1986; **80**: 12-19.

57. National Malaria Control Programme – Zimbabwe *Strategy for Zimbabwe 2008 – 2013*. Ministry of Health and Child Welfare, Zimbabwe, October 2008.

58. Global Fund – Zimbabwe. Zimbabwe Global Fund Round 10 Proposal Form, 2010. URL: www.theglobalfund.org/documents/rounds/10/(accessed February, 2012).

59. Mastbaum O. Past and present of malaria position of malaria in Swaziland. *J Trop Med & Hyg* 1957a; **60**: 119-127.

60. Ministry of Health and Social Welfare. *Malaria control in Swaziland*. National Malaria Control Programme, Mbabane, Kingdom of Swaziland, 1999.

61. Fontaine RE. Review of the Swaziland malaria control program conducted between March 30 – April 25, 1987. Vector Biology and Control Project, Medical Service Consultants Inc., AR-052,1987.

62. Kunene S, Phillips AA, Gosling RD, Kandula D, Novotny JM. A national policy for malaria elimination in Swaziland: a first of sub-Saharan Africa. *Malar J* 2011; **10**: 313.

63. Lunde TM, Bayoh MN, Lindtjørn B. How malaria models relate temperature to malaria transmission *Parasites & Vectors* 2013; **6**: 20.

64. Gething PW, van Boeckel TP, Smith DL, Guerra CA, Patil AP,*et al*. Modelling the global constraints of temperature on transmission of *Plasmodium falciparum* and *P. vivax*. *Parasite & Vectors* 2011; **4**: 92.

65. Shililu JI, Grueber WB, Mbogo CM, Githure JI, Riddiford LM,*et al*. Development and survival of *Anopheles gambiae* eggs in drying soil: influence of the rate of drying, egg age, and soil type. *J Am Mosq Control Assoc* 2004; **20**: 243-247.

66. Gray EM, Bradley TJ. Physiology of desiccation resistance in *Anopheles gambiae* and *Anopheles arabiensis*.*Am J Trop Med & Hyg* 2005; **73**: 553-559.

67. MODIS Reprojection Tool Release 4.1. Land Processes Distributed Active Archive Center (LP DACC), U.S. Geological Survey Earth Resources Observation & Science Center and South Dakota School of Mines & TechnologyNASA FTP server ftp://e4ftl01u.ecs.nasa.gov (Accessed July 25,2011).

68. Guerra CA, Snow RW, Hay SI. Defining the global limits of malaria transmission in 2005. *Adv Parasitol* 2006; **62**: 157-179.

69. Guerra CA, Gikandi PW, Tatem AJ, Noor AM, Smith DL,*et al*. The limits and intensity of *Plasmodium falciparum*: implications for malaria control and elimination worldwide. *PLoS Medicine* 2008, **5**: e38.

70. Linard C, Gilbert M, Tatem AJ. Assessing the use of global land cover data for guiding large area population distribution modelling. *GeoJournal* 2010;**76**: 525–538.

71. Linard C, Gilbert M, Snow RW, Noor AM, Tatem AJ. Population distribution, settlement Patterns and accessibility across Africa in 2010. *PLoS One* 2012*,* **7**: e31743.

72. The Afripop Project. www.afripop.org. (Accessed July 18, 2013).

73. Mennis J. Dasymetric mapping for estimating population in small areas. *Geography Compass* 2009; **3**: 727-745

74. United Nations Population Division World *Urbanization Prospects: The 2011 Revision* (United Nations, New York). Available at: http://esa.un.org/unup/; accessed March 2013.

75. Global Rural-Urban Mapping Project (GRUMP) http://sedac.ciesin.columbia.edu/gpw; (accessed Mar 10, 2013).

76. United Nations World Population Prospects. http://esa.un.org/wpp/ (Accessed July 18, 2013).

77. Metselaar D & van Thiel PH. Classification of malaria. *Trop Geogr Med* 1959; **11**: 157–161.

78. Macdonald G, Göeckel GW. The malaria parasite rate and interruption of transmission. *Bull World Health Org* 1964; **31**: 365–377.

79. Lysenko AJ, Semashko IN . *Geography of malaria. A medico-geographic profile of an ancient disease* [in Russian]. In: Lebedew AW, editor. Moscow: *Acad Sci* USSR; 1968. p. 25-146.

80. PubMed. National Center for Biotechnology Information. Available: (http:// www.ncbi.nlm.nih.gov/sites/entrez (accessed Jan 10, 2013).

81. Armed Forces Pest Management Board http://www.afpmb.org/publications.htm; (accessed Feb, 2012).

82. World Health Organization Library Database http://www.who.int/library (accessed March, 2012).

83. Institute de Recherché pour le Developmenthttp://www.ird.fr (accessed Jan, 2013).

84. African Journals Online: (accessed Jan, 2013).

85. Malaria World http://www.malaria-world.com(accessed Jan 10, 2013).

86. Roll Back Malaria news alert service http://www.rollbackmalaria.org/newsalert (accessed Jan, 2013).

87. Environmental Health at USAID http://www.ehproject.org (accessed Jan 10, 2013).

88. Santé Tropicale http://www.santetropicale.com (accessed Jan 10, 2013).

89. HINARI http://www.who.int/hinari (accessed Jan, 2013).

90. Snow RW, Marsh K, le Sueur D. The need for maps of transmission intensity to guide malaria control in Africa. *Parasitol Today* 1996; **12**: 455-457.

91. MARA - Malaria Risk in Africa. Available: http://www.mara-database.org (accessed April 12, 2012).

92. RBM MERG http://www.rbm.who.int/mechanisms/merg.html (accessed Jan 14, 2013).

93. Corsi DJ, Neuman M, Finlay JE, Subramanian S. Demographic and health surveys: a profile. *Int J Epi* 2012; **41**: 1602-1613.

94. MEASURE-ICF programme http://www.measuredhs.com/Data/ (accessed Jan 14, 2013).

95. Médecins sans Frontières http://www.msf.org (accessed Jan, 2013).

96. MERLIN http://www.merlin.org.uk (accessed Jan 12, 2013).

97. MENTOR http://thementorinitiative.org (accessed Jan 12, 2013).

98. Carter Center http://www.cartercenter.org (accessed Jan 12, 2013).

99. *Food Security and Nutrition Analysis Unit* http://www.fsnau.org (accessed May 5, 2012).

100. Shape Consulting http://cms.shapeconsulting.org (accessed Jan, 2013).

101. Malaria Consortium http://www.malariaconsortium.org (accessed Jan, 2013).

102. Noor AM, Alegana VA, Patil AP, Moloney G, Borle M, *et al*. Mapping the receptivity of malaria risk to plan the future of control in Somalia. *BMJ Open* 2012; **2**: e001160.

103. The Kenya Medical Research Institute-Wellcome Trust Research programme. Malaria Public Health Department collaborators. http://www.kemri-wellcome.org/index.php/en/group_page/12#

104. O'Meara WP, Barcus M, Wongsrichanalai C, Muth S, MaguireJD, *et al*. Readertechnique as a source of variability in determining malaria parasite density by microscopy. *Malar J* 2006; **5**: 118.

105. Gitonga CW, Kihara JH, Njenga SM, Awundo K, Noor AM, *et al*. Use of rapid diagnostic tests in malaria school surveys in Kenya: does under-performance matter for planning malaria control? *Am J Trop Med & Hyg* 2012; **87**: 1004-1011.

106. World Health Organization-Foundation for Innovative New Diagnostics. *Malaria Rapid Diagnostic Test Performance. Results of WHO product testing of malaria RDTs: Round 4*. http://apps.who.int/iris/bitstream/10665/77748/1/9789241504720_eng.pdf. (Accessed Jul 16, 2013).

107. Rennie W, Phetsouvanh R, Lupisan S, Vanisaveth V, Hongvanthong B, *et al*. Minimizing human error in malaria rapid diagnosis: clarity of written instructions and health worker performance. *Trans R Soc Trop Med & Hyg* 2007; **101**: 9–18.

108. Harvey SA, Jennings L, Chinyama M, Masaninga F, Mulholland K, *et al*.Improving community health worker use of malaria rapid diagnostic tests in Zambia: package instructions, job aid and job aid-plus-training. *Malar J* 2008; **7**: 160.

109. Okell LC, Ghani AC, Lyons E,Drakeley CJ. Sub-microscopic infection in *Plasmodium falciparum* endemic populations: a systematic review and meta-analysis. *J Infect Dis* 2009; **200**: 1509–1517.

110. Gething PW, Patil AP, Smith DL, Guerra CA, Elyazar IR, *et al*. A new world malaria map: *Plasmodium falciparum* endemicity in 2010. *Malar J* 2011; **10**: 378.

111. GEOnet Names http://www.earth-info.nga.mil/gns/html/cntry_files.html (accessed Jan, 2013).

112. Falling Rain Genomics' Global Gazetteer http://www.fallingrain.com (accessed Jan, 2013).

113. Alexandria Digital Library http://www.alexandria.ucsb.edu (accessed Jan, 2013).

114. Hill LL. Core elements of digital gazetteers: Placenames, categories, and footprints. *Research & Advanced Technology for Digital Libraries, Proceedings* 2000; **1923**:280-290.

115. FAO (2008). The Global Administrative Unit Layers (GAUL). EC-FAO Food Security Programme, Food and Agriculture Organization, United Nations.

116. Lehner B, Doll P. Development and validation of a global database of lakes, reservoirs and wetlands. *J Hydrol* 2004; **296**: 1-22.

117. Gregory RD, Blackburn TM. Parasite prevalence and host sample size. *Parasitol Today* 1991; **7**: 316-318.

118. Jovani R,Tella JL.Parasite prevalence and sample size: misconceptions and solutions. *Trends Parasitol* 2006; **22**: 214-218.

119. Mirghani ES, Nour BYM, Bushra SM, Hassan El I, Snow RW, *et al.* The spatial-temporal clustering of *Plasmodium falciparum* infection over eleven years in Gezira State, The Sudan. *Malar J* 2010; **9**: 172.

120. Noor AM, Elmardi KA, Tarig A, Patil AP, Amine AAA, *et a*l. Malaria risk mapping for control in the Republic of Sudan. *Am J Trop Med & Hyg* 2012; **87**: 1012-1021.

121. Pull JH, Grab B. A simple epidemiological model for evaluating the malaria inoculation rate and the risk of infection in infants. *Bull World Health Org* 1974; **51**: 507-516.

122. Smith DL, Guerra CA, Snow RW, Hay SI. Standardizing estimates of malaria prevalence. *Malar J* 2007; **6**: 131.

123. Babyak MA. What you see may not be what you get: a brief, nontechnical introduction to overfitting in regression-type models. *Psychosom Med* 2004; **66**: 411-421.

124. Harrell FE Jr. Regression modeling strategies: with applications to linear models, logistic regression and survival analysis. New York: Springer, 2001.

125. Craig M H, Sharp B L, Mabaso M L, Kleinschmidt I. Developing a spatial-statistical model and map of historical malaria prevalence in Botswana using a staged variable selection procedure.*Int J Health Geogr* 2007; **6**: 44.

126. Murtaugh, PA. Performance of several variable-selection methods applied to real ecological data. Ecology Letters 2009; **12**: 1061-1068.

127. Raffalovich, L.E., Deane, G.D., Armstrong D. &Tsao, H.S. Model selection procedures in social research: Monte- Carlo simulation results. *J Appl Stat* 2008; **35**: 1093–1114.

128. Molineaux L: The epidemiology of human malaria as an explanation of its distribution, including some implications for its control. Malaria: Principles and Practice of Malariology. W.Wernsdorfer and I. McGregor. London, Churchill Livingstone. 2: 913-998, 1988.

129. Snow RW, Gilles HM: *The epidemiology of malaria*. In: Warrel DA, Gilles HM editors. Bruce-Chwatt's essential malariology. 4th ed. London: Arnold, 2002.

130. Scharlemann J, Benz D, Hay S, Purse B, Tatem A, *et al.* Global data for ecology and epidemiology: a novel algorithm for temporal Fourier processing MODIS data. *PLoS One* 2008; **9**:e1408.

131. Hijmans R, Cameron S, Parra J, Jones P, Jarvis A. Very high resolution interpolated climate surfaces for global land areas. *Int J Clim* 2005*;* **25**: 1965-1978.

132. Bayoh MN, Lindsay SW. Effect of temperature on the development of the aquatic stages of Anopheles gambiaesensustricto (Diptera: Culicidae). *Bull Entomol Res* 2003; **93**: 375-81.

133. Bayoh MN, Lindsay SW. Temperature-related duration of aquatic stages of the Afrotropical malaria vector mosquito Anopheles gambiae in the laboratory. *Med Vet Entomol* 2004; **18**: 174-179.

134. Muirhead-Thompson RC. Mosquito behaviour in relation to malaria transmission and control in the tropics. London, Edward Arnold & Co, 1951.

135. Kirby MJ, Lindsay SW. Responses of adult mosquitoes of two sibling species, *Anopheles arabiensis*and *A. gambiaes.s*. (Diptera: Culicidae), to high temperatures. *Bull Entomol Res* 2004*;* **94**: 441-8.

136. WorldClim. http://www.worldclim.org/download.htm. (accessed Dec 7, 2012).

137. Gill CA . The role of meteorology in malaria. *Indian J Med Res* 1920*;* **8**: 633-693.

138. Dutta HM, Dutt AK. Malaria ecology: a global perspective. *Soc Sci and Med* 1978; **12**: 69-84.

139. Noor AM, Gething PW, Alegana VA, Patil AP, Hay SI, *et al*. The risks of malaria infection in Kenya in. *BMC Infect Dis* 2009; **9**: 180.

140. NASA Earth Observatory: http://earthobservatory.nasa.gov/Features/MeasuringVegetation/measuring_vegetation_4.php. (Accessed Jan 17, 2013).

141. Trape, JF, Zoulani A. Malaria and urbanization in Central Africa: the example of Brazzaville. Part II: results of entomological surveys and epidemiological analysis. *Trans R Soc Trop Med Hyg* 1987; **81**: 10–18.

142. Omumbo JA, Guerra CA, Hay SI, Snow RW.The influence of urbanisation on measures of *Plasmodium falciparum* infection prevalence in East Africa. *Acta Trop* 2005; **93**: 11-21.

143. Wang SJ, Lengeler C, Smith TA, Vounatsou P, Diadie DA, *et al*. Rapid urban malaria appraisal (RUMA) I: epidemiology of urban malaria in Ouagadougou. *Malar J* 2005; **4**: 43.

144. Hay SI, Guerra CA, Tatem AJ, Atkinson PM, Snow RW. Urbanization, malaria transmission and disease burden in Africa. *Nat Rev Microbiol* 2005; **3:** 81-90.

145. Tatem AJ, Guerra CA, Kabaria CW, Noor AM, Hay SI. Human population, urban settlement patterns and their impact *Plasmodium falciparum* malaria endemicity. *Malar J* 2008; **7**: 218.

146. Balk D, Pozzi F, Yetman G, Deichmann U, Nelson A. The distribution of people and the dimension of place: Methodologies to improve global population estimates in urban and rural areas. New York CIESIN, Columbia University, 2004.

147. Elvidge C, Baugh K, Kihn E, Koehl H, Davis E. Mapping city lights with nighttime data from the DMSP Operational Linescan System. *Photogramm Eng Rem S* 1997; **63**: 727-734.

148. Derksen S, Keselman HJ (1992). Backward, forward and stepwise automated subset selection algorithms: frequency of obtaining authentic and noise variables. *Br J Math Stat Psych* 1992; **45**: 265–82.

149. Peduzzi PN, Concato J, Kemper E, Holford TR, *et al*. A simulation study of the number of events per variable in logistic regression analysis. *J Clin Epidemiol* 1996; **49**: 1373–9.

150. Hoeting JA, Davis RA, Merton AA, Thompson SE. Model selection for geostatistical models. *Ecol Appl* 2006; **16**: 87-88.

151. Miller A. *Subset Selection in Regression*. Boca Raton, FL: Chapman & Hall,2002.

152. Lumley T .*Leaps: regression subset selection (R package) Version 2.7*. 2010.

153. Mcleod AI, Xu C. Bestglm: Best Subset GLM 2011. http://cran.r-project.org/web/packages/bestglm/vignettes/bestglm.pdf (accessed Mar 10, 2013).

154. Diggle, P, Ribeiro Jr, P . Model-based Geostatistics. Springer,2007.

155. Chilés J and Delfiner P. Geostatistics: Modeling Spatial Uncertainty. New York: Wiley, 1999.

156. Hay SI, Sinka ME, Tatem AJ, Patil AP, Guerra CA, et al. Developing global maps of the dominant Anopheles vectors of human malaria. *PLoS Med* 2009; **7**: e1000209.

157. Gilks W, Richardson S, Spiegelhalter D. Markov chain Monte Carlo in practice. London: Chapman & Hall, 1996.

158. Rue H, Martino S, Chopin N, (2009). Approximate Bayesian inference for latent Gaussian model by using integrated nested Laplace approximations (with discussion). *J. R. Stat. Soc.* B 2009; **71:** 319–392.

159. Cameletti M, Lindgren F, Simpson D, Rue H. Spatio-temporal modeling of particulate matter concentration through the spde approach. *Adv Stat Analy* 2012; 1-23.

160. Rue H, Held L. Gaussian Markov Random Fields. Vol. 104 of Monographs on Statistics and Applied Probability. Chapman & Hall/CRC, 2005.

161. Lindgren F, Rue H, Lindström J. An explicit link between Gaussian fields and Gaussian Markov random fields: the stochastic partial differential equation approach (with discussion). *J. R. Stat. Soc. B* 2011; **73(4):** 423–498.

162. Bolin D, Lindgren F (2011). Spatial models generated by nested stochastic partial differential equations, with an application to global ozone mapping. *Ann Appl Stat* 2011; **5 (1)**: 523-550.

163. Simpson D, Lindgren F, Rue H. In order to make spatial statistics computationally feasible, we need to forget about the covariance function. *Environmetrics* 2012a*;* **23 (1)**: 65-74.

164. Simpson D, Lindgren F, Rue H. Think continuous: Markovian Gaussian models in spatial statistics. *Spat Stat* 2012b; **1**: 16-29.

165. Lindgren F, Rue H (2013). *Bayesian Spatial and Spatio-temporal Modelling with R-INLA.* http://www.math.ntnu.no/inla/r-inla.org/papers/jss/lindgren.pdf. (accessed July 12, 2013).

166. Daly C, Neilson R, Phillips D. A statistical-topographic model for mapping climatological precipitation over mountainous terrain. *J Appl Meteorol* 1994; **33 (2)**: 140-158.

167. Isaacs E, Srivastava R .*Appl geost*. Oxford*: Oxford* University Press, 1989.

168. World Health Organization. *World Malaria Report for 2012.* WHO, Geneva. http://www.who.int/malaria/publications/world_malaria_report_2012/en/index.html. Accessed 15 July 2013.

169. Human Development Index 2013. http://hdr.undp.org/en/media/HDR2013_EN_Summary.pdf. Accessed 19 November 2013.
